# Microbiome Analysis Reveals the Dynamic Alternations in Gut Microbiota of Diarrheal *Giraffa camelopardalis*

**DOI:** 10.3389/fvets.2021.649372

**Published:** 2021-05-28

**Authors:** Li Xi, Yumin Song, Xinxi Qin, Jincheng Han, Yung-Fu Chang

**Affiliations:** ^1^Department of Animal Science, Shangqiu Normal University, Shangqiu, China; ^2^Henan Engineering Research Center of Development and Application of Green Feed Additives, Shangqiu, China; ^3^Linyi Agricultural Science and Technology Career Academy, Linyi, China; ^4^Department of Population Medicine and Diagnostic Sciences, College of Veterinary Medicine, Cornell University Ithaca, NY, United States

**Keywords:** gut microbiota, diarrhea, 16S rRNA, giraffe, sequencing

## Abstract

The ruminant gut microbial community's importance has been widely acknowledged due to its positive roles in physiology, metabolism, and health maintenance. Diarrhea has been demonstrated to cause adverse effects on gastrointestinal health and intestinal microecosystem, but studies regarding diarrheal influence on gut microbiota in *Giraffa camelopardalis* have been insufficient to date. Here, this study was performed to investigate and compare gut microbial composition and variability between healthy and diarrheic *G. camelopardalis*. The results showed that the gut microbial community of diarrheal *G. camelopardalis* displayed a significant decrease in alpha diversity, accompanied by distinct alterations in taxonomic compositions. Bacterial taxonomic analysis indicated that the dominant bacterial phyla (*Proteobacteria, Bacteroidetes*, and *Firmicutes*) and genera (*Escherichia Shigella* and *Acinetobacter*) of both groups were the same but different in relative abundance. Specifically, the proportion of *Proteobacteria* in the diarrheal *G. camelopardalis* was increased as compared with healthy populations, whereas *Bacteroidetes, Firmicutes, Tenericutes*, and *Spirochaetes* were significantly decreased. Moreover, the relative abundance of one bacterial genus (*Comamonas*) dramatically increased in diarrheic *G. camelopardalis*, whereas the relative richness of 18 bacterial genera decreased compared with healthy populations. Among them, two bacterial genera (*Ruminiclostridium_5* and *Blautia*) cannot be detected in the gut bacterial community of diarrheal *G. camelopardalis*. In summary, this study demonstrated that diarrhea could significantly change the gut microbial composition and diversity in *G. camelopardalis* by increasing the proportion of pathogenic to beneficial bacteria. Moreover, this study first characterized the distribution of gut microbial communities in *G. camelopardalis* with different health states. It contributed to providing a theoretical basis for establishing a prevention and treatment system for *G. camelopardalis* diarrhea.

## Introduction

*Giraffa camelopardalis* is the largest known ruminant native to the African continent (1). They have distinct long necks and legs, off-white skin patches separated by white-off white color, and a pair of ossicones (horn-like structure) on their head. Microbiota of the gastrointestinal tract plays an important role in digestion in ruminants and *G. camelopardalis* (2–4). This gut microbiota, particularly in the rumen, plays vital roles in gut homeostasis, host immunity, and physiology (5–7). Any disturbance in the balance of this microbiota can lead to digestive ailments resulting in diarrhea, weakness, regurgitation of the ingesta, or immunosuppression that may pose adverse effects on the heath of the host (8–10). There is little literature available on diarrhea in *G. camelopardalis*. The non-infectious causes may include simple indigestion due to ingestion of rotten feed (during drought conditions), and infectious causes are reported to be of the parasitic, viral, and/or bacterial origin (11, 12).

Diarrhea in the neonates could be fatal, as it may lead to excessive secretion of water and nutrients from the body leading to a negative energy balance, starvation, weakness, and death (13, 14). In adults, it may lead to loss of appetite, submissive behavior, and even lack of fertility (15, 16). It may also lead to the contamination of the environment posing a threat to the other healthy animals of the herds and even to the different species of the animals living in close contact with the animal of interest or the environment (17, 18). In wild animals, veterinary health services are not readily available; hence, it is important to know the cause of intestinal disorders to predict and prepare possible treatment regimens.

In recent years, a lot has been performed to study gut microbiota in humans and its effects on body pathophysiology and psychology. The interest in microbiomes has recently extended further into studies centered on domestic and wild animals and even some key invertebrate lines (19, 20). In humans, reduced microbial diversity has long been linked to diarrhea (14). Hence, critical knowledge and practices to maintain diversity are indispensable. It has been revealed that the microbiome in mammals is dependent upon the host environment, host species, type of feed, and host health conditions (21, 22). As a lot of gut microbiota reside in the forestomach of the ruminants, which act in a variety of ways, e.g., symbiotic, commensal, or parasitic/pathogenic relationship, some of them do live as opportunistic pathogens (23, 24). Forehand knowledge may help draw strategies, conclusions, and therapy regimens based on the microbiota's species makeup (25). Genome sequencing technologies have made it easier and faster than before to analyze the species of such environments (26, 27). Our study aimed to evaluate the type of microbiota species and the difference and type of microbiota species in the diarrheic *G. camelopardalis* of a Chinese zoo.

## Materials and Methods

### Sample Acquisition

In this study, the *G. camelopardalis* used for sample acquisition inhabited in the Linyi Zoological and Botanical Gardens (Linyi, China), including five diarrheal *G. camelopardalis* and five healthy *G. camelopardalis* (~2 years old). The ratio of females to males in both groups was 3:2. All the selected animals were raised under the same conditions and received the same immunization. Sufficient water and feed were provided *ad libitum* for all animals throughout the entire experiment. One day before sample collection, all the *G. camelopardalis* were placed in a dedicated area of the Zoological and Botanical Gardens and maintained a normal diet. Five separate fresh fecal samples, ~10 g, were achieved from each individual using a sterile tool the following morning. Afterward, the collected samples were resampled from the intermediate areas to minimize contamination by bedding and flooring. All the collected feces were immediately stored in the sterilized plastic tubes, snap-frozen utilizing liquid nitrogen, and stored at −80°C for further study.

### DNA Extraction and Illumine MiSeq Sequencing

Bacterial genomic DNA was extracted using QIAamp DNA Mini Kit (QIAGEN, Hilden, Germany) based on the manufacturer's instructions. The genomic DNA was subjected to quality assessment by using 0.8% (w/v) agarose gel electrophoresis, whereas its concentration was quantified *via* utilizing ultraviolet–visible spectrophotometer (NanoDrop 2000, United States). The target sequence reflecting the composition and diversity of the microbial ribosomal RNA was used as targets, and corresponding primers were synthesized according to the conservative regions in the sequence. The obtained primers (338F: ACTCCTACGGGAGGCAGCA and 806R: GGACTACHVGGGTWTCTAAT) were added to specific adaptors and then applied to amplify the V3/V4 regions. Polymerase chain reaction (PCR) amplification was conducted in triplicates to guarantee the accuracy of the results. After the PCR reaction, the amplified products were subjected to quality detection using 2% agarose gel electrophoresis, and a gel recovery kit (Axygen, CA, USA) was used for recovering the target fragments. Recovered PCR products were fluorescently quantified on a microplate reader (BioTek, FLx800) as per the preliminary quantitative results of electrophoresis. Subsequently, each sample was mixed in corresponding proportion according to the sequencing quality requirements. The purified PCR products were used to construct the sequencing library *via* using Illumina TruSeq (Illumina, United States) following manufacturer's specifications. Before the sequencing, the libraries were performed the quality evaluation and fluorescence quantification, and the libraries with only one peak and concentration > 2 nM were considered qualified. The final libraries were diluted and mixed in proportion and subjected to 2 × 300 bp paired-end sequencings using the MiSeq sequencing machine.

### Bioinformatics and Data Analysis

The paired-end sequences achieved from sequencing were merged into a tag, and the Trimmomatic software (v0.33) was applied to screen the qualified raw reads. Afterward, the Cutadapt software (1.9.1) was used to identify and remove primer sequences to obtain high-quality reads. The high-quality reads of each sample were performed splice and chimera removal using FLASH software (v1.2.7) and UCHIME (v4.2), respectively, to obtain effective reads. The obtained effective reads were clustered and operational taxonomic unit (OTU) distinction according to 97% similarity, and the most abundant sequence in each OTU was selected as the representative sequence. The representative sequence of each OTU was taxonomically classified on the basis of the Ribosomal Database Project database. The representative sequence of each OTU was compared with the template sequence of the corresponding database to obtain the taxonomic information. Successive analyses of alpha diversity and beta diversity were performed based on the normalized output date. Alpha diversity was calculated based on the richness distribution of OTUs in different samples. Meanwhile, beta diversity was conducted utilizing QIIME (version 1.7.0) to analyze the difference and similarity among different samples. Moreover, the rarefaction and rank curves were generated to assess the sequencing depth, richness, and evenness. The linear discriminant analysis effect size was generated to investigate the differentially abundant taxon. SPSS statistical program (v18.0) and GraphPad Prism (version 5.0c) were applied to statistical analysis. The criterion of significance was conducted at *P* < 0.05, and data were presented as means ± SD.

## Results

### Sequences Analyses

**Figure 1:**
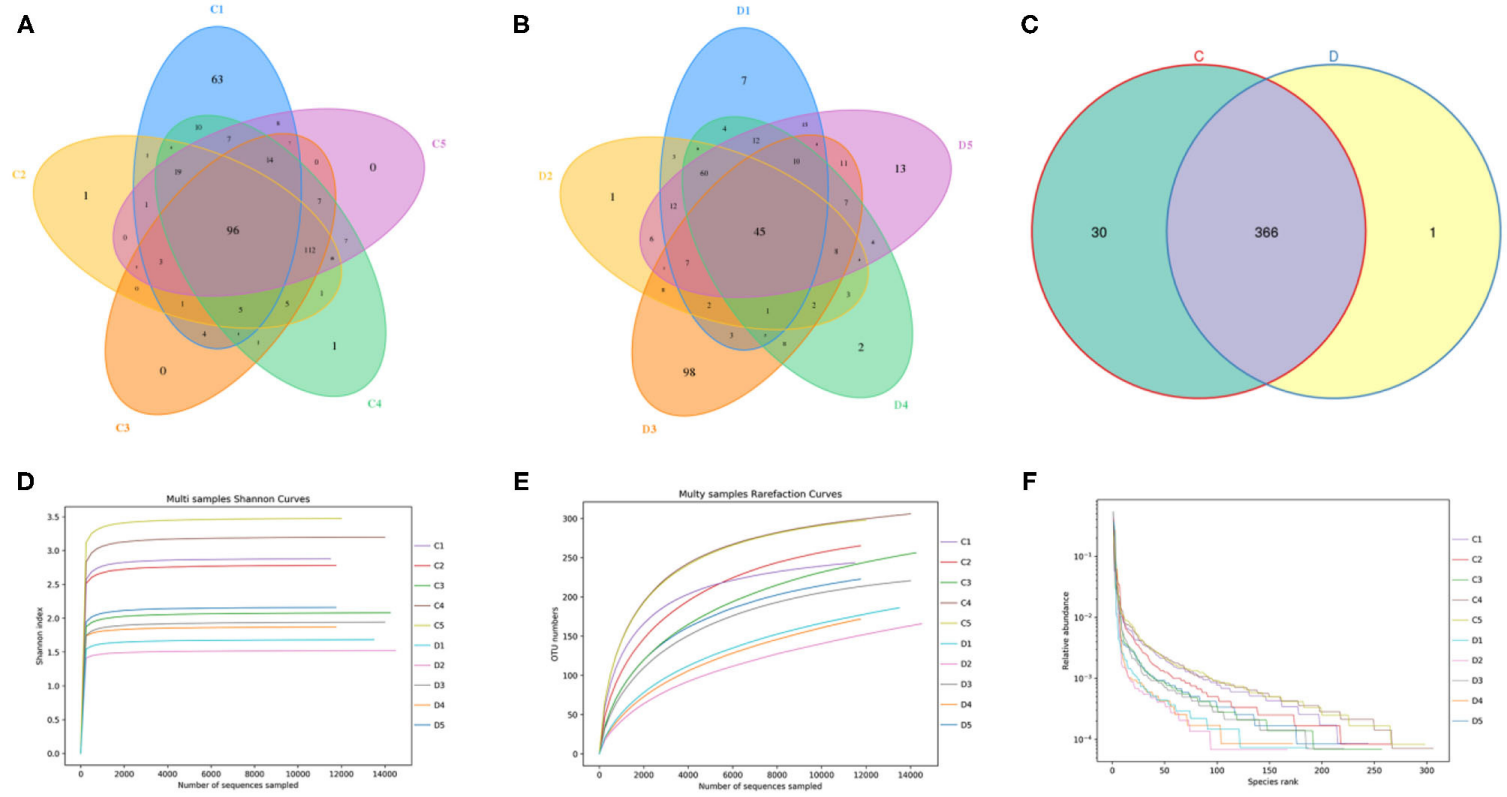
Venn diagrams and sample feasibility analysis between both groups. **(A,B)** Venn diagrams for the bacterial OTU distribution in each sample of control and diarrhea groups. **(C)** Venn diagrams showing independent and shared bacterial OTUs in control and diarrhea groups. **(D,E)** Bacterial rarefaction curves were applied to evaluate the Sequencing depth. Different colored lines distinguished each individual. **(F)** Rank Abundance curve.

In this investigation, 10 fecal samples collected from *G. camelopardalis* were subjected to amplicon sequencing, and a total of 93,903 and 87,922 raw sequences were obtained from the C and D groups, respectively (Table 1). After quality control processing and eliminating the unqualified data, 148,166 high-quality reads were achieved from all the samples, with an average of 14,816 (ranging from 13,185 to 16,010) reads per sample. After taxonomic assignment, a total of 763 OTUs (C=396, D=367) were identified based on 97% nucleotide-sequence similarity. Additionally, the core OTUs in the C and D groups were 96 and 45, respectively (Figures 1A,B). The Venn diagram showed that there were 366 OTUs shared from all the samples, accounting for ~47.97% of the total OTUs (Figure 1C). The Shannon curve of each sample was relatively flat and showed a saturated tendency when the number of qualified sequences was more than 2,000, suggesting that the sequencing quantity and depth met the requirement for further analysis (Figures 1D,E). Furthermore, the rank abundance curve in each sample of both groups was wide and falling relaxedly and almost parallel to the x-axis, displaying excellent abundance and evenness (Figure 1F).

**Table 1:** Sequence information of each sample.

**Sample**	**Raw reads**	**Clean reads**	**Effective reads**	**AvgLen (bp)**	**Effective (%)**
C1	19,354	16,291	15,628	1,465	80.75
C2	17,931	14,725	14,121	1,467	78.75
C3	19,451	16,456	15,912	1,464	81.81
C4	19,055	16,312	16,010	1,462	84.02
C5	18,112	15,033	14,576	1,467	80.48
D1	18,143	15,339	14,915	1,461	82.21
D2	19,484	16,456	15,991	1,462	82.07
D3	17,826	14,671	14,359	1,464	80.55
D4	16,135	13,553	13,185	1,461	81.72
D5	16,334	13,810	13,469	1,461	82.46

### Alterations in Gut Microbial Diversities With the Effect of Diarrhea

**Figure 2:**
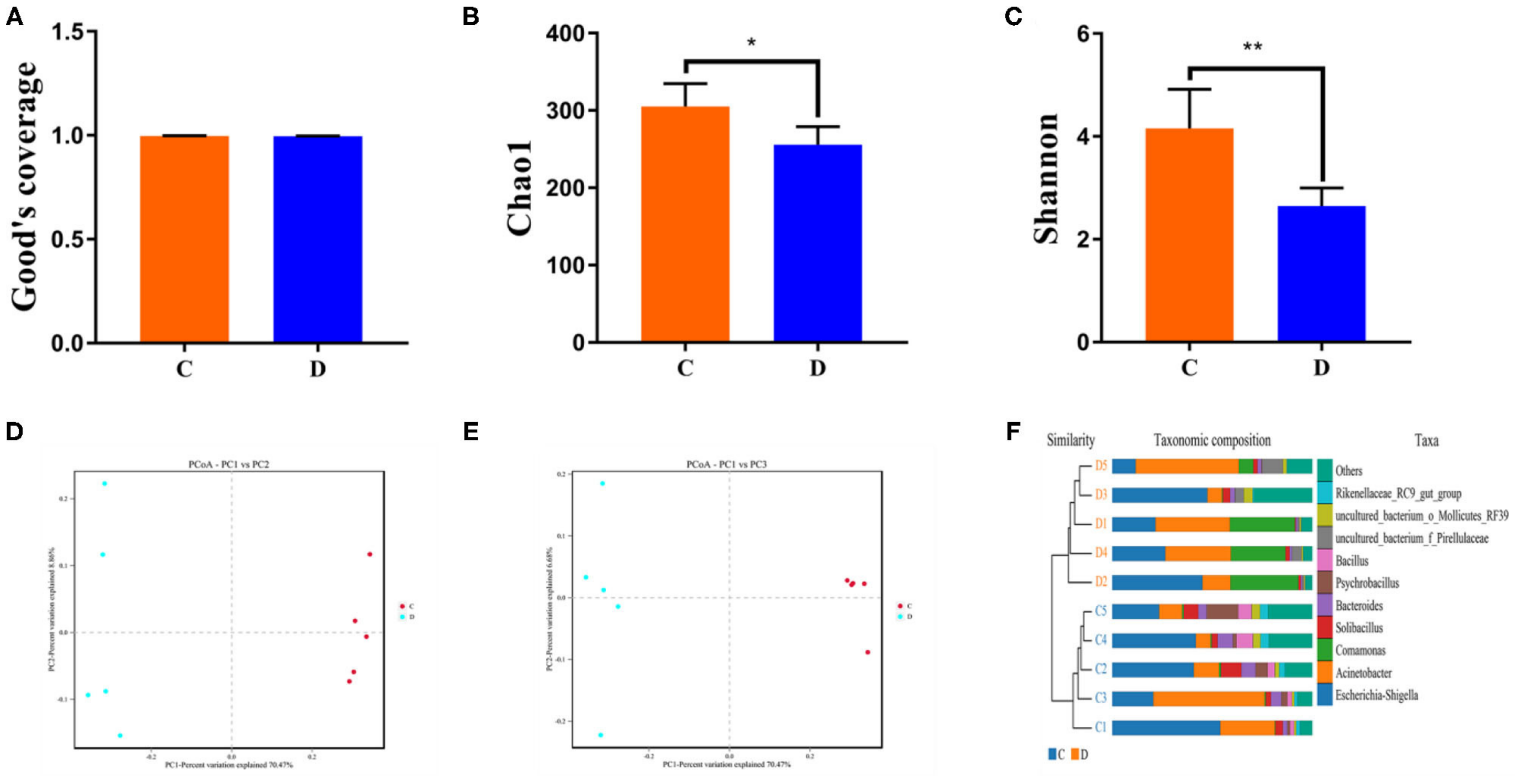
Gut microbial alpha and beta diversities analysis. Gut bacterial alpha diversity can be determined by Good's coverage **(A)** and Chao **(B)** and Shannon **(C)** indices. **(D,E)** Principal coordinate analysis of gut microbial community in control and diarrhea groups. Points with same color represent samples of the same group. **(F)** Clustering analysis on the basis of unweighted pair-group method with arithmetic means. Data are expressed as the mean ± SD. ^*^P < 0.05, ^**^P < 0.01.

To further investigate the dynamics of gut bacterial community diversities in both groups, the qualified sequences from the sequencing were aligned to calculate alpha-diversity indices, including Good's coverage and Chao1 and Simpson indices. Good's coverage estimates varied from 99.4 to 99.7% of the species for all samples, indicating excellent coverage (Figure 2A). The average of the Chao1 index in the control group varied from 268.17 to 349.33, whereas the Simpson index ranged from 0.68 to 0.90. Intergroup analysis of alpha diversity intuitively revealed that there were statistically significant differences in the Chao1 (305.04 ± 29.67 vs. 255.81 ± 23.33, *p* = 0.019) and Shannon (4.16 ± 0.76 vs. 2.65 ± 0.35, *p* = 0.004) indices between the control and diarrhea groups, indicating that diarrhea significantly decreased the gut microbial abundance and diversity of *G. camelopardalis* (Figures 2B,C). Both the weighted and the unweighted principal coordinates analysis plots, which reflect the difference and similarity between groups and individuals, were generated to evaluate the bacterial beta diversity (Figures 2D,E). The beta diversity analysis revealed that the individuals in the control group were clustered together and separated from the diarrhea group, which was in line with the unweighted pair-group method with arithmetic mean analysis results, indicating a distinct difference in the principal compositions of gut bacterial community between both groups (Figure 2F).

### Significant Alterations in the Gut Bacterial Taxonomic Compositions With the Effect of Diarrhea

**Figure 3:**
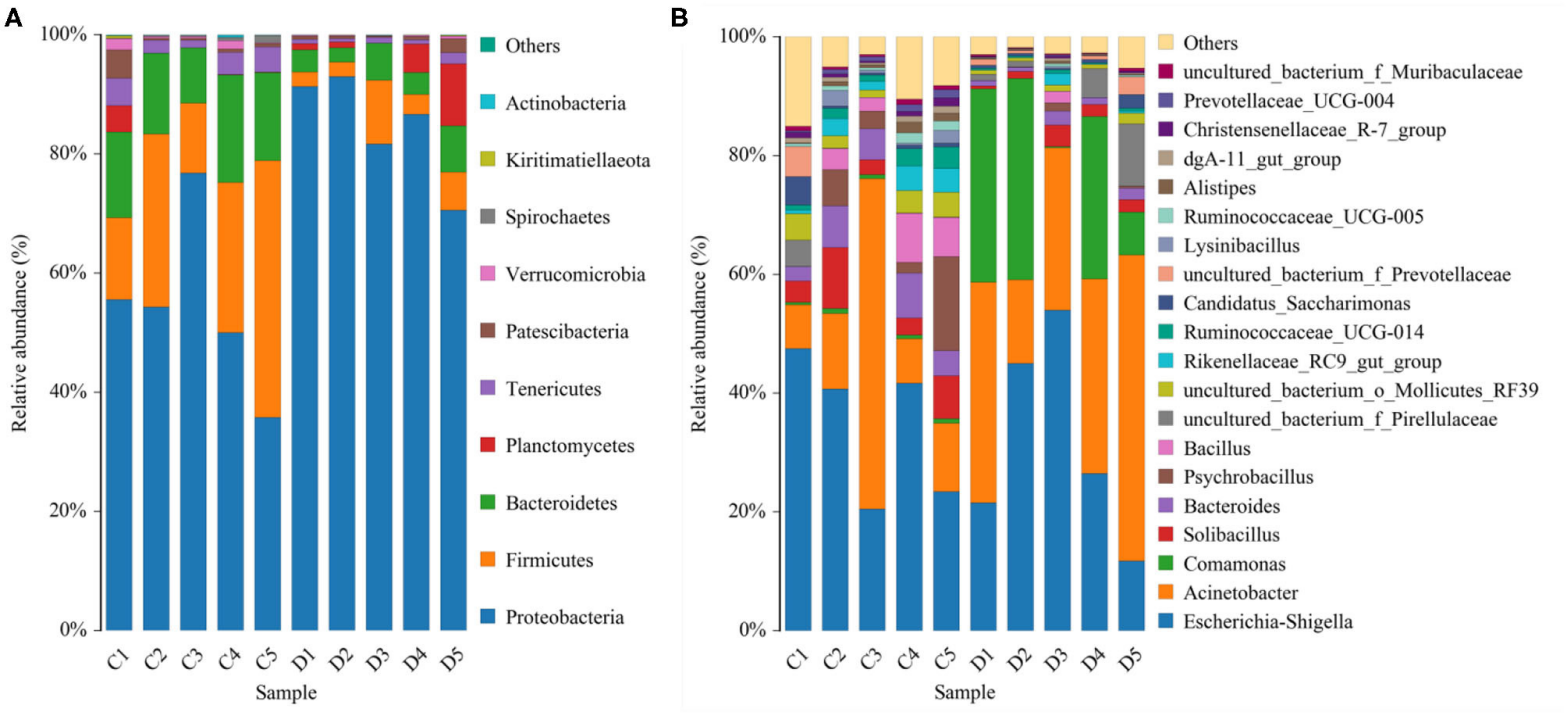
Proportional distributions of gut microbial phyla and genera identified in healthy and diarrheal *Giraffa camelopardalis*. Color blocks with different lengths represent relative abundance of each bacterial taxon. **(A,B)** indicated taxa assignments at the levels of phylum (top 10) and genus (top 20).

**Figure 4:**
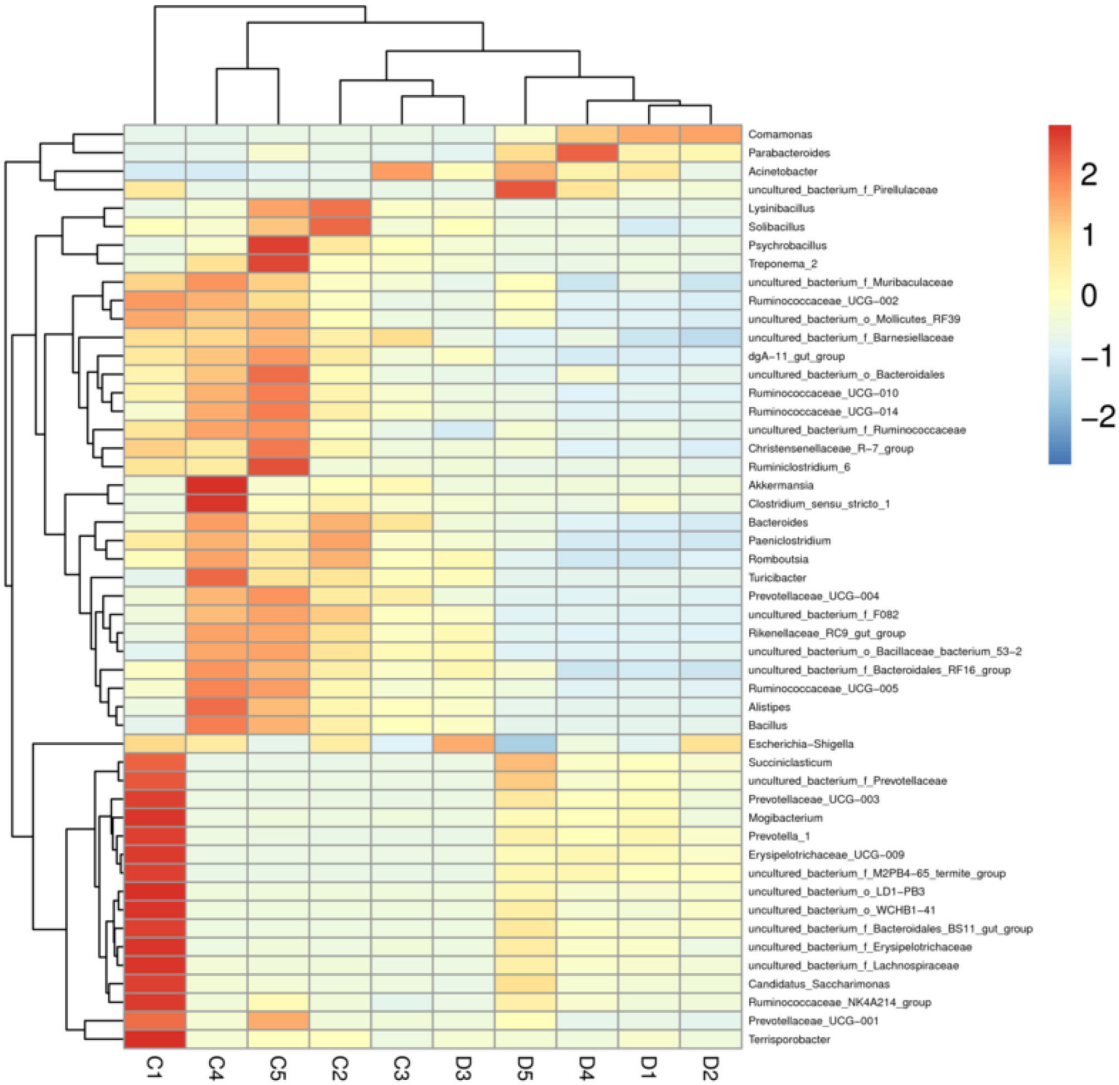
Heatmap of relative abundance of each bacterial genus (top 50). Relative abundance of bacterial genus was reflected by color intensity.

The proportions of preponderant taxa at the levels of phylum and genus were evaluated *via* microbial taxon assignment in both groups. We observed considerable variability in gut bacterial taxonomic compositions. As shown in Figure 3, a total of 13 phyla were identified from the 10 samples, ranging from 9 to 13 phyla per sample. According to the phylum assignment results, *Proteobacteria* (55.15, 85.07%), *Firmicutes* (24.20, 5.06%), and *Bacteroidetes* (13.97, 4.68%) were the three most dominant phyla in control and diarrhea groups, respectively, which accounted for more than 90% of the taxonomic groups identified (Figure 3A). Other phyla such as *Kiritimatiellaeota* (0.08, 0.04%), *Verrucomicrobia* (0.78, 0.19%), *Spirochaetes* (0.48, 0.04%), and *Actinobacteria* (0.11, 0.01%) in both groups, respectively, were represented with a lower abundance. At the genus level, *Escherichia Shigella* (34.36%) is the most prevalent bacteria in the *G. camelopardalis* of the control group followed by the *Acinetobacter* (20.00%) and *Bacteroides* (5.36%), which together made up 59.73% of the total 16S ribosomal RNA gene sequences. However, *Escherichia Shigella* (32.80%), *Acinetobacter* (31.74%), and *Comamonas* (20.47%) were observed as the predominant in the diarrheal *G. camelopardalis* with slightly different from the control populations (Figure 3B). Moreover, the distribution of bacterial genera in each sample could also be observed in the heatmap (Figure 4).

**Figure 5:**
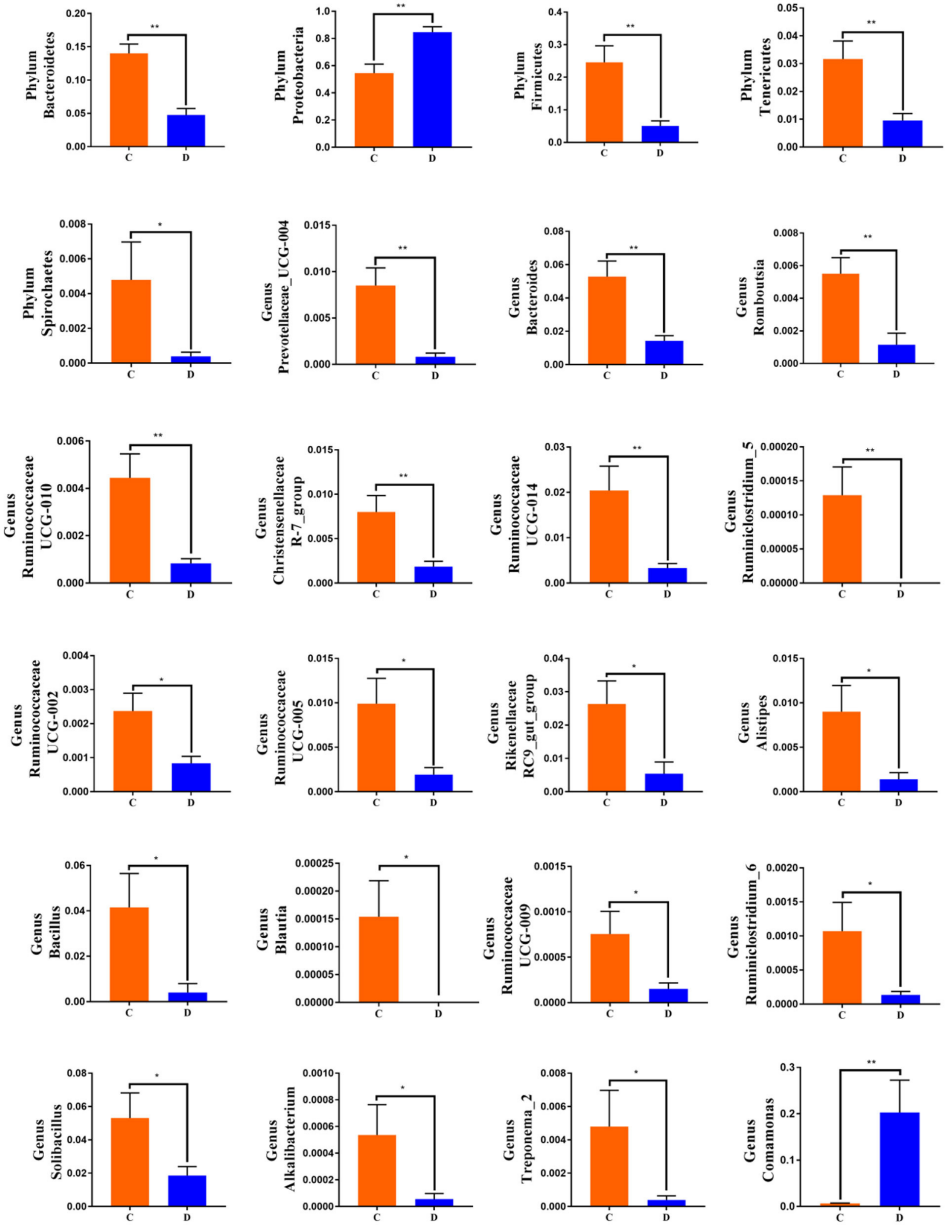
Significant shifts in gut microbial abundance at phylum and genus levels in both groups. Data are expressed as mean ± SD. ^*^P < 0.05, ^**^P < 0.01.

**Figure 6:**
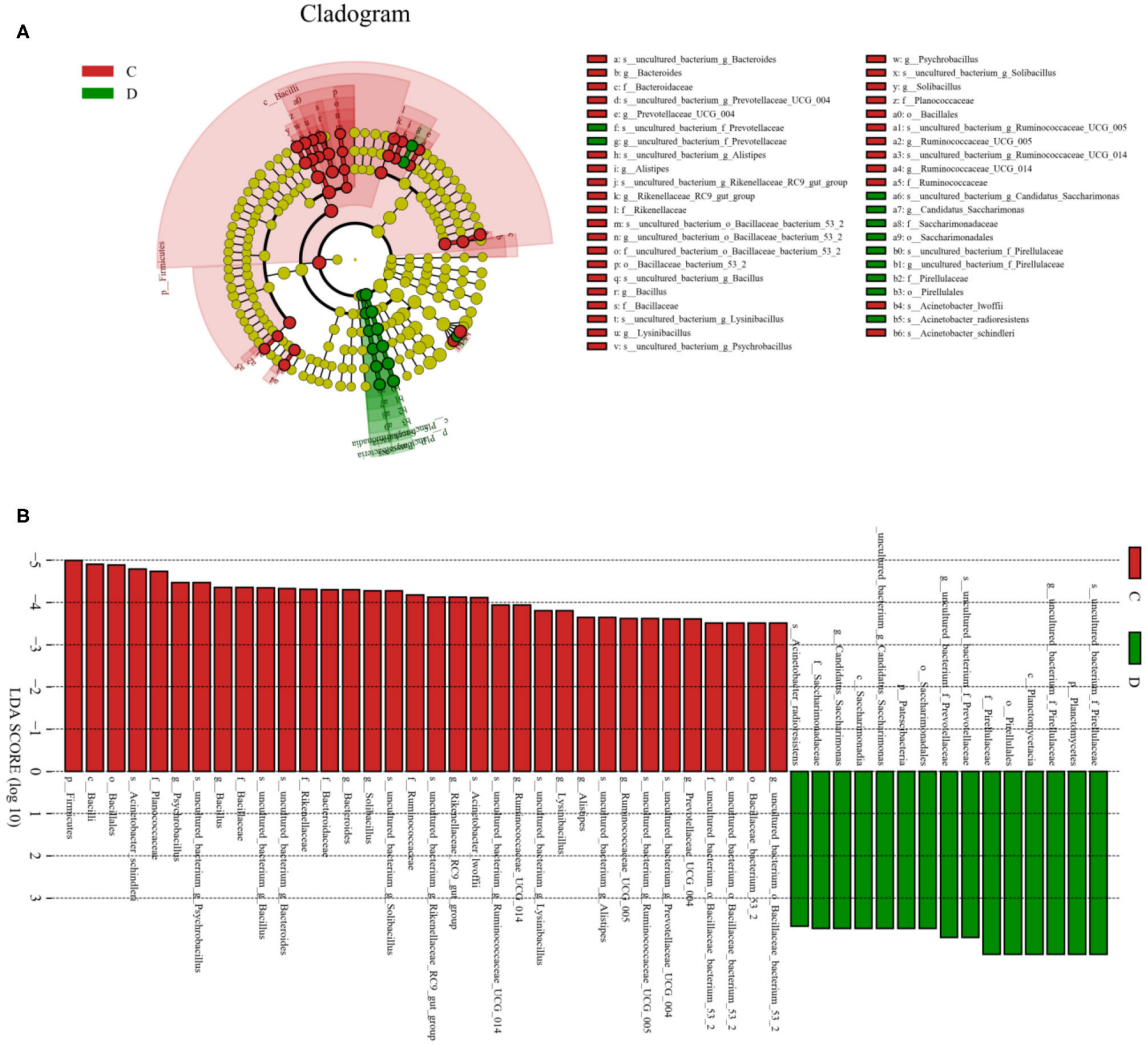
Integrated linear discriminant analysis effect size analysis and linear discriminant analysis (LDA) indicated differences in relative abundance of both groups. **(A)** Cladogram revealing phylogenetic distribution of gut bacterial community associated with control and diarrhea groups. Yellow circles represented taxa with no obvious differences. **(B)** LDA scores displaying the distinct bacterial difference between control and diarrhea groups. LDA scores >3.6 were considered statistically significant.

To further investigate the differences in taxonomic compositions of *G. camelopardalis* in the different health states, Metastats analysis was performed for different classification levels (Figure 5). Results revealed that at the phylum level, the abundance of *Proteobacteria* in diarrheal *G. camelopardalis* was significantly dominant than control populations, whereas the *Bacteroidetes, Firmicutes, Tenericutes*, and *Spirochaetes* were lower (*P* < 0.05 or *P* < 0.01). Additionally, 19 genera were identified to be significantly different between both groups. Of these discriminatory taxa, the relative abundances of 18 bacterial genera (*Prevotellaceae_UCG-004, Bacteroides, Romboutsia, Ruminococcaceae_UCG-010, Christensenellaceae_R-7_group, Ruminococcaceae_UCG-014, Ruminiclostridium_5, Ruminococcaceae_UCG-002, Ruminococcaceae_UCG-005, Rikenellaceae_RC9_gut_group, Alistipes, Bacillus, Blautia, Ruminococcaceae_UCG-009, Ruminiclostridium_6, Solibacillus, Alkalibacterium*, and *Treponema_2*) dramatically decreased, whereas the relative abundance of one bacterial genus (*Comamonas*) observably increased with the effect of diarrhea. Furthermore, two bacterial genera (*Ruminiclostridium_5* and *Blautia*) cannot be detected in the gut microbiota of diarrheal *G. camelopardalis*. Given this discriminant analysis cannot distinguish the primary taxon, linear discriminant analysis effect size analysis coupled with linear discriminant analysis was performed to identify the specific bacteria associated with diarrhea (Figure 6). Besides those significantly different bacteria mentioned earlier, we also observed that several bacteria such as *Planctomycetes, Patescibacteria*, and *Candidatus_Saccharimonas* were markedly overrepresented in the feces of diarrheal *G. camelopardalis*, whereas *Lysinibacillus* and *Psychrobacillus* were the most preponderant microbiota in the control group.

### Correlation Network Analysis

**Figure 7:**
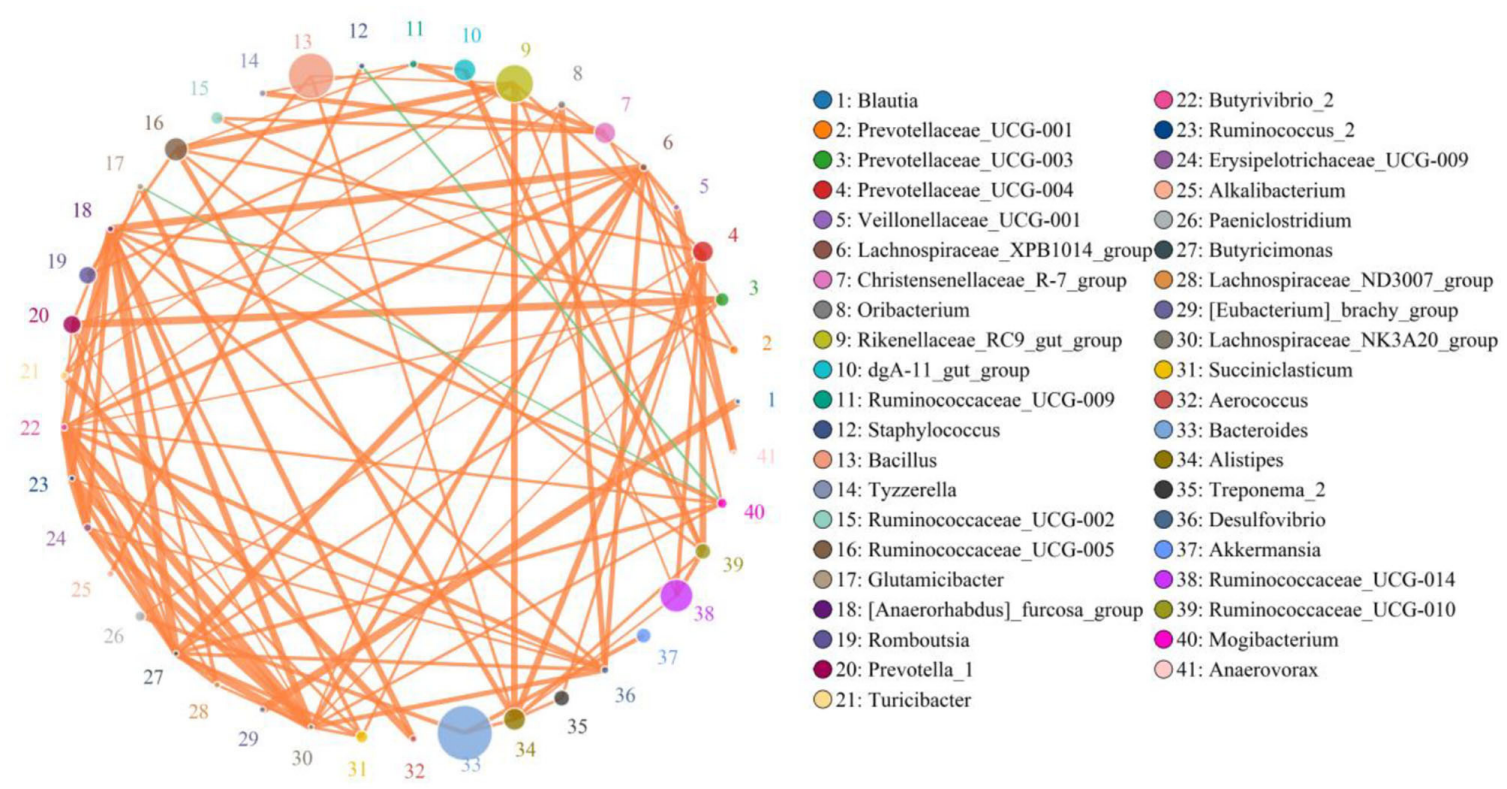
Network analysis revealing potential correlation between different bacterial genera. Color and size of circles represent bacterial genera name and abundance, respectively. Correlative strength between both bacteria genera can be evaluated by thickness of lines. Green and orange lines indicate negative and positive correlation, respectively.

Network analysis was conducted using a Python program to investigate linkages among different bacterial genera in an intestinal microbial community. The top 41 most correlated genera are displayed in Figure 7. This network graph consists of 1,006 edges and 73 nodes. *Bacteroides* was positively associated with *Paeniclostridium* (0.9515), *Alistipes* (0.9394), *Treponema_2* (0.7781), and *Akkermansia* (0.9515) (Supplementary Table 1). *Ruminococcaceae_UCG-014* was positively related to *Alistipes* (0.9273), *Ruminococcaceae_UCG-010* (0.9515), and *Prevotellaceae_UCG-004* (0.9515). *Ruminococcaceae_UCG-005* was positively related to *Ruminococcaceae_UCG-010* (0.9394), *Ruminococcaceae_UCG-009* (0.9273), *Prevotellaceae_UCG-004* (0.9394), and *Alistipes* (0.9636). *Prevotellaceae_UCG-004* was positively associated with *Paeniclostridium* (0.9273), *Ruminococcaceae_UCG-010* (0.9758), and *Treponema_2* (0.9362). *Christensenellaceae_R-7_group* was positively correlated with *Ruminococcaceae_UCG-009* (0.9636), *Tyzzerella* (0.9636), *Ruminococcaceae_UCG-002* (0.9394), and *Prevotellaceae_UCG-001* (0.9394). *Rikenellaceae_RC9_gut_group* was positively associated with *Ruminococcaceae_UCG-005* (0.9758), *Romboutsia* (0.9394), *Turicibacter* (0.9245), *Prevotellaceae_UCG-004* (0.9394), *Ruminococcaceae_UCG-010* (0.9394), and *Alistipes* (0.9758). *Ruminococcaceae_UCG-009* was positively associated with *Tyzzerella* (0.9273). *Bacillus* was positively associated with *Turicibacter* (0.9405), *Alistipes* (0.9380), and *Rikenellaceae_RC9_gut_group* (0.9255). *Alistipes* was positively associated with *Treponema_2* (0.9362), *Prevotellaceae_UCG-004* (0.9758), and *Ruminococcaceae_UCG-010* (0.9394).

## Discussion

Ruminant gut microbiota involving trillions of microbes is a complicated and interactive ecosystem that plays vital roles in metabolism, immunity, nutrient absorption, and intestinal mucosal barrier maintenance. Moreover, the evidence demonstrated that the gut microbial community was also a crucial barrier for the host against the invasion and colonization of foreign pathogens, implying its crucial roles in the prevention and amelioration of diseases (28). Diarrhea is widely prevalent in various animals, which is considered an important factor causing the reduction of global animal productivity (29). Currently, many measures have been conducted to prevent diarrhea, but it still presently displays a high prevalence rate. Research into gut microbiota has recently revealed its essential role in developing diarrhea (30, 31). Therefore, investigating the gut microbial composition and structure is of great significance for preventing and treating diarrhea. Currently, research into a mammalian gut bacterial community has covered many species, such as goat, sheep, yak, and cattle, but few reports have been published on the differences of gut microbiota in *G. camelopardalis* with different health states. Taking advantage of this gap, we systematically investigated the gut microbial shifts in health and diarrheal *G. camelopardalis*. Results indicated a significant difference in gut microbial composition and diversity between *G. camelopardalis* with different health states.

Given the particularity of this species, we selected feces as the research object to evaluate the composition and structure of a gut bacterial community. This study revealed a dramatically decreased alpha diversity in the gut microbiota of diarrheal *G. camelopardalis*, indicating its intestinal flora imbalance. Moreover, principal coordinates analysis revealed that the samples of the control group were clustered together and separated from the diarrheal samples, suggesting an obvious difference in the primary composition of the gut microbiota between control and diarrhea groups. Previous studies have demonstrated that gut microbial diversity and abundance were positively associated with intestinal function, and the higher microbial abundance and diversity in the intestine contribute to energy utilization and perform complex physiological functions (32). However, we observed a significant decrease in the gut microbial diversity of diarrheal *G. camelopardalis*, indicating intestinal dysfunction. The mammalian gut bacterial community was normally affected by species, disease, and diet during development and reached stability at maturity (33, 34). Although the gut microbial community is often in flux, its function can remain stable because of many functionally redundant species (35). The stabilized gut bacterial community is the prerequisite for the host against the invasion of pathogenic bacteria and performs various biological functions, whereas obvious alternations in the microbial community affect its physiological function and threaten the host's health (36, 37). Accumulating evidence revealed that intestinal flora alternation may result in impaired intestinal barrier function and decreased immunity, which in turn increased the susceptibility to pathogenic bacteria (38, 39). Therefore, some opportunistic pathogens may also show pathogenicity in diarrheal *G. camelopardalis* with significantly altered gut microbiota, which significantly increases the morbidity of a host.

This study revealed that *Proteobacteria, Firmicutes*, and *Bacteroidetes* were the three most preponderant phyla in the gut microbial community of all samples. Consistent with previous studies on other mammals, those phyla were also observed to be abundantly presented in the intestines of goat, cattle, yak, and sheep, suggesting their importance in intestinal ecology and function (40–42). Interestingly, although diarrhea did not alter the diversity of dominant bacteria phyla in *G. camelopardalis*, the proportion of some intestinal bacteria changed significantly. At the phylum level, the percentage of *Proteobacteria* in the gut microbiota of diarrheal *G. camelopardalis* increased, whereas the ratio of *Bacteroidetes, Firmicutes, Tenericutes*, and *Spirochaetes* decreased as compared with control populations. *Firmicutes* are mainly responsible for decomposing cellulose, whereas *Bacteroidetes* have been demonstrated to play a vital role in digesting carbohydrates and proteins and benefit the maturation of the intestinal immune system (43, 44). Therefore, the higher abundance of *Firmicutes* and *Bacteroidetes* in a gut microbial community may contribute to meet the host's daily high energy and nutritional demands (43). Moreover, most members of *Firmicutes* were regarded as beneficial bacteria, which contribute to improving the intestinal environment and against pathogenic invasion (45, 46). *Proteobacteria* consisting of many gram-negative bacteria, including *Salmonella, Helicobacter pylori, Vibrio cholerae*, and *Escherichia coli*, is the largest bacterial phylum (47, 48). Some members of the phylum *Proteobacteria* are common opportunistic pathogens and pathogenic bacteria, which can cause diarrhea, gastritis, gastrointestinal ulcers, and even death, posing a significant threat to animal health (49, 50). Those results indicated a significant alteration in the dominant bacteria phyla of diarrheal *G. camelopardalis*, which further implied its gut microbiota imbalance.

Importantly, this study also observed a high variation in some bacterial genera in both groups, and this variation may play key roles in the intestinal ecosystem and function. Interestingly, the relative abundances of 18 bacterial genera with distinct differences displayed a tendency to decline, and two bacterial genera cannot be detected in the gut microbial community of diarrheal *G. camelopardalis*, suggesting that these bacterial genera could not adapt to the intestinal environments found in ill hosts. We speculated that diarrhea results in the deterioration of the gut environment, where the growth of mutualistic bacterial clades is limited. Remarkably, most of these significantly decreased bacteria (*Prevotellaceae_UCG-004, Bacteroides, Ruminococcaceae_UCG-010, Christensenellaceae_R-7_group, Ruminococcaceae_UCG-014, Ruminiclostridium_5, Ruminococcaceae_UCG-002, Ruminococcaceae_UCG-005, Rikenellaceae_RC9_gut_group, Ruminococcaceae_UCG-009*, and *Ruminiclostridium_6*) are regarded as potential beneficial bacteria in the intestine. *Prevotellaceae* has been demonstrated to display the characteristics of digesting high carbohydrate food, pectin, and hemicellulose (51). *Bacteroides*, a vital anaerobic genus, play a fundamental role in an intestinal ecosystem through decomposing polysaccharides (52). Therefore, the higher abundances of *Prevotellaceae* and *Bacteroides* in the gut bacterial community contribute to more energy intake and meet the host's energy demand during growth. *Ruminococcaceae*, mainly colonizing in the caecum and colon, showed the ability to degrade cellulose and starch (53). *Christensenellaceae* can secrete multiple hydrolases such as β-glucosidase, β-galactosidase, and α-arabinosidase, indicating an important role in feed efficiency (54). Decreased bacterial loads of the mutualistic species discussed earlier are closely related to digestive decomposition, which may be why diarrheal animals are accompanied by reduced body weight and digestive ability. Moreover, *Ruminococcaceae* is involved in the positive regulation of the immune system and intestinal environment closely related to healthy homeostasis (55, 56). Recent studies on *Ruminococcaceae* have provided evidence that its abundance in the gut environment was negatively associated with liver cirrhosis, non-alcoholic fatty liver, and increased intestinal permeability (57, 58). *Ruminiclostridium* was previously reported to produce short-chain fatty acids, which contributed to maintaining functionality and morphology of intestinal epithelial cells and the regulation of gut microbiota balance (59, 60). Moreover, short-chain fatty acids regulate energy intake through the brain–gut axis to alleviate the development of obesity and diabetes and change the gastrointestinal tract's pH against the proliferation of pathogens (61, 62). *Rikenellaceae*, a key intestinal beneficial bacterium, displays multiple physiological functions, such as degrading plant-derived polysaccharides and limit inflammation by stimulating T-regulatory cell differentiation (63, 64). *Comamonas* was the only bacteria with increased abundance in diarrheal *G. camelopardalis*, which has been demonstrated to result in life-threatening bacteremia (65).

Disrupted gut microbial communities have long been demonstrated to be the pathological mediators of many diseases (66, 67). Microorganisms such as bacteria, fungi, protozoa, and viruses in the intestine interact in a synergistic, antagonistic, or symbiotic relationship to form a stable intestinal environment (68). Hence, shifts in the relative abundances of some bacteria in the intestine may affect the other bacterial functions, aggravating the gut microbiota alteration. The correlation network analysis indicated a clear positive correlation among those significantly changed bacterial genera, which may further weaken this bacterial function and affect the holistic intestinal function. Furthermore, we also observed that *Akkermansia* was positively correlated with the decreased *Bacteroides*, indicating that the function of this bacterium may be affected. Studies have demonstrated that *Akkermansia* could decrease the risk of obesity, inflammation, diabetes, and associated complications *via* regulating the metabolism and maintaining gastrointestinal health (69). Moreover, aside from improving intestinal barrier function and mucosal immunity, *Akkermansia* could also enhance the antitumor effect of cisplatin in mice with lung cancer (69, 70). This study conveyed a vital message that diarrhea directly altered gut microbial composition and diversity and affected the other bacterial functions through the interactions between bacteria, which may further result in the alternations of intestinal function in *G. camelopardalis*.

In conclusion, this study first investigated the effect of diarrhea on the gut microbial community of *G. camelopardalis*. Results indicated that the gut bacterial community in diarrheal *G. camelopardalis* undergoes significant alterations, characterized by decreased microbial diversity and increased proportion of harmful bacteria. These results also expanded the understanding of gut microbial characteristics in *G. camelopardalis* and conveyed a crucial message that gut microbiota alteration may be one of the reasons for the occurrence or exacerbation of diarrhea. However, several limitations in the current study need to be noticed, including sampling methods, individual variation, and relatively smaller size.

## Data Availability Statement

The datasets presented in this study can be found in online repositories. The names of the repository/repositories and accession number(s) can be found at: https://www.ncbi.nlm.nih.gov/, PRJNA689292.

## Ethics Statement

The animal study was reviewed and approved by the Laboratory Animal Research Center of Henan province in China and the Animal Welfare and Ethics Committee of the Shangqiu Normal University (Permit No. 41230576532).

## Author Contributions

LX conceived, designed the experiments, and wrote the manuscript. YS contributed sample collection and reagent preparation. XQ analyzed the data. JH and YC revised the manuscript. All authors reviewed the manuscript.

## Conflict of Interest

The authors declare that the research was conducted in the absence of any commercial or financial relationships that could be construed as a potential conflict of interest.
