# Supplementary material for: Microbiome Analysis Reveals the Dynamic Alternations in Gut Microbiota of Diarrheal Giraffa camelopardalis
Source: Front Vet Sci. 2021 May 28;8:649372. doi: 10.3389/fvets.2021.649372 (PMC8192810; doi:10.3389/fvets.2021.649372)
Supplement: Supplementary file 1 [file Table_1.pdf]

Supplementary Table 1 Edge property analysis among different bacterial genera

Source	Target	weight	color
**Escherichia-Shigella**	Acinetobacter	0.6727	negative
**Acinetobacter**	dgA-11_gut_group	0.7091	negative
**Acinetobacter**	Christensenellaceae_R-7_group	0.6364	negative
**Acinetobacter**	Terrisporobacter	0.697	negative
**Acinetobacter**	Ruminococcaceae_UCG-009	0.6727	negative
**Acinetobacter**	Family_XIII_AD3011_group	0.6565	negative
**Acinetobacter**	Ruminiclostridium_5	0.7647	negative
**Comamonas**	Solibacillus	0.6848	negative
**Comamonas**	Bacteroides	0.6606	negative
**Comamonas**	Rikenellaceae_RC9_gut_group	0.6727	negative
**Comamonas**	Ruminococcaceae_UCG-005	0.7212	negative
**Comamonas**	Alistipes	0.6606	negative
**Comamonas**	dgA-11_gut_group	0.697	negative
**Comamonas**	Christensenellaceae_R-7_group	0.6364	negative
**Comamonas**	Romboutsia	0.6848	negative
**Comamonas**	Ruminococcaceae_UCG-002	0.697	negative
**Comamonas**	Parabacteroides	0.8667	positive
**Comamonas**	Paeniclostridium	0.6364	negative
**Comamonas**	Ruminiclostridium_6	0.7939	negative
**Comamonas**	Ruminococcaceae_UCG-009	0.7091	negative

**Comamonas**	Enterococcus	0.6606	negative
**Comamonas**	Glutamicibacter	0.64	negative
**Comamonas**	Oscillibacter	0.7333	negative
**Comamonas**	Stenotrophomonas	0.676	negative
**Solibacillus**	Bacteroides	0.7455	positive
**Solibacillus**	Psychrobacillus	0.7455	positive
**Solibacillus**	Bacillus	0.6753	positive
**Solibacillus**	Rikenellaceae_RC9_gut_group	0.7939	positive
**Solibacillus**	Ruminococcaceae_UCG-014	0.8182	positive
**Solibacillus**	Lysinibacillus	0.7212	positive
**Solibacillus**	Ruminococcaceae_UCG-005	0.8303	positive
**Solibacillus**	Alistipes	0.7697	positive
**Solibacillus**	dgA-11_gut_group	0.8182	positive
**Solibacillus**	Christensenellaceae_R-7_group	0.7697	positive
**Solibacillus**	Prevotellaceae_UCG-004	0.7939	positive
**Solibacillus**	Romboutsia	0.8667	positive
**Solibacillus**	Ruminococcaceae_UCG-010	0.8061	positive
**Solibacillus**	Treponema_2	0.7477	positive
**Solibacillus**	Ruminococcaceae_UCG-002	0.6606	positive
**Solibacillus**	Paeniclostridium	0.8667	positive
**Solibacillus**	Ruminiclostridium_6	0.697	positive
**Solibacillus**	Turicibacter	0.7306	positive

**Solibacillus**	Ruminococcaceae_UCG-009	0.8303	positive
**Solibacillus**	Enterococcus	0.7697	positive
**Solibacillus**	Aerococcus	0.7176	positive
**Solibacillus**	Glutamicibacter	0.64	positive
**Solibacillus**	Tyzzerella	0.6727	positive
**Solibacillus**	Tyzzerella_4	0.7176	positive
**Solibacillus**	Ruminiclostridium_5	0.7511	positive
**Bacteroides**	Psychrobacillus	0.7455	positive
**Bacteroides**	Bacillus	0.8567	positive
**Bacteroides**	Rikenellaceae_RC9_gut_group	0.8788	positive
**Bacteroides**	Ruminococcaceae_UCG-014	0.8909	positive
**Bacteroides**	Lysinibacillus	0.6364	positive
**Bacteroides**	Ruminococcaceae_UCG-005	0.8788	positive
**Bacteroides**	Alistipes	0.9394	positive
**Bacteroides**	dgA-11_gut_group	0.7818	positive
**Bacteroides**	Christensenellaceae_R-7_group	0.7091	positive
**Bacteroides**	Prevotellaceae_UCG-004	0.9152	positive
**Bacteroides**	Romboutsia	0.8788	positive
**Bacteroides**	Clostridium_sensu_stricto_1	0.7576	positive
**Bacteroides**	Ruminococcaceae_UCG-010	0.8667	positive
**Bacteroides**	Treponema_2	0.8511	positive
**Bacteroides**	Akkermansia	0.9515	positive

**Bacteroides**	Ruminococcaceae_UCG-002	0.6848	positive
**Bacteroides**	Succiniclasticum	0.6688	negative
**Bacteroides**	Paeniclostridium	0.9515	positive
**Bacteroides**	Ruminiclostridium_6	0.6727	positive
**Bacteroides**	Turicibacter	0.8469	positive
**Bacteroides**	Ruminococcaceae_UCG-009	0.7818	positive
**Bacteroides**	Enterococcus	0.8424	positive
**Bacteroides**	Aerococcus	0.8211	positive
**Bacteroides**	Glutamicibacter	0.7823	positive
**Bacteroides**	Oscillibacter	0.6727	positive
**Bacteroides**	Tyzzerella	0.6727	positive
**Bacteroides**	Alkalibacterium	0.7379	positive
**Bacteroides**	[Eubacterium]_brachy_group	0.8057	positive
**Bacteroides**	Staphylococcus	0.7629	positive
**Bacteroides**	Oxalobacter	0.6338	positive
**Bacteroides**	Parvibacter	0.6636	positive
**Bacteroides**	Blautia	0.8057	positive
**Bacteroides**	Ruminiclostridium_5	0.6964	positive
**Psychrobacillus**	Bacillus	0.8942	positive
**Psychrobacillus**	Rikenellaceae_RC9_gut_group	0.8424	positive
**Psychrobacillus**	Ruminococcaceae_UCG-014	0.8788	positive
**Psychrobacillus**	Lysinibacillus	0.8788	positive

**Psychrobacillus**	Ruminococcaceae_UCG-005	0.7455	positive
**Psychrobacillus**	Alistipes	0.8303	positive
**Psychrobacillus**	dgA-11_gut_group	0.7333	positive
**Psychrobacillus**	Prevotellaceae_UCG-004	0.8303	positive
**Psychrobacillus**	Prevotella_1	0.7091	negative
**Psychrobacillus**	Romboutsia	0.7818	positive
**Psychrobacillus**	Clostridium_sensu_stricto_1	0.6727	positive
**Psychrobacillus**	Ruminococcaceae_UCG-010	0.8061	positive
**Psychrobacillus**	Treponema_2	0.7781	positive
**Psychrobacillus**	Prevotellaceae_UCG-003	0.7212	negative
**Psychrobacillus**	Succiniclasticum	0.816	negative
**Psychrobacillus**	Paeniclostridium	0.7333	positive
**Psychrobacillus**	Mogibacterium	0.6688	negative
**Psychrobacillus**	Erysipelotrichaceae_UCG-009	0.8469	negative
**Psychrobacillus**	Turicibacter	0.8211	positive
**Psychrobacillus**	Oribacterium	0.7379	negative
**Psychrobacillus**	Lachnospiraceae_XPB1014_group	0.7435	negative
**Psychrobacillus**	Desulfovibrio	0.7306	negative
**Psychrobacillus**	Aerococcus	0.7693	positive
**Psychrobacillus**	Glutamicibacter	0.7047	positive
**Psychrobacillus**	Butyrivibrio_2	0.7952	negative
**Psychrobacillus**	Alkalibacterium	0.7754	positive

**Psychrobacillus**	[Eubacterium]_brachy_group	0.8467	positive
**Psychrobacillus**	[Anaerorhabdus]_furcosa_group	0.7823	negative
**Psychrobacillus**	Staphylococcus	0.8004	positive
**Psychrobacillus**	Lachnospiraceae_ND3007_group	0.6896	negative
**Psychrobacillus**	Tyzzerella_4	0.7693	positive
**Psychrobacillus**	Ruminococcus_2	0.8469	negative
**Psychrobacillus**	Lachnospiraceae_NK3A20_group	0.7952	negative
**Psychrobacillus**	Butyricimonas	0.7823	negative
**Psychrobacillus**	Blautia	0.8467	positive
**Psychrobacillus**	Ruminococcaceae_UCG-013	0.6487	negative
**Psychrobacillus**	Corynebacterium_1	0.7178	positive
**Psychrobacillus**	Ruminiclostridium_5	0.635	positive
**Bacillus**	Rikenellaceae_RC9_gut_group	0.9255	positive
**Bacillus**	Ruminococcaceae_UCG-014	0.8817	positive
**Bacillus**	Lysinibacillus	0.7691	positive
**Bacillus**	Ruminococcaceae_UCG-005	0.8692	positive
**Bacillus**	Alistipes	0.938	positive
**Bacillus**	dgA-11_gut_group	0.7441	positive
**Bacillus**	Prevotellaceae_UCG-004	0.8817	positive
**Bacillus**	Prevotella_1	0.6378	negative
**Bacillus**	Romboutsia	0.8567	positive
**Bacillus**	Clostridium_sensu_stricto_1	0.8254	positive

**Bacillus**	Ruminococcaceae_UCG-010	0.8254	positive
**Bacillus**	Treponema_2	0.8499	positive
**Bacillus**	Akkermansia	0.7691	positive
**Bacillus**	Prevotellaceae_UCG-003	0.6503	negative
**Bacillus**	Succiniclasticum	0.8609	negative
**Bacillus**	Paeniclostridium	0.8004	positive
**Bacillus**	Mogibacterium	0.747	negative
**Bacillus**	Erysipelotrichaceae_UCG-009	0.8338	negative
**Bacillus**	Turicibacter	0.9405	positive
**Bacillus**	Oribacterium	0.7097	negative
**Bacillus**	Ruminococcaceae_UCG-009	0.7191	positive
**Bacillus**	Lachnospiraceae_XPB1014_group	0.8004	negative
**Bacillus**	Desulfovibrio	0.8004	negative
**Bacillus**	Aerococcus	0.9005	positive
**Bacillus**	Glutamicibacter	0.8471	positive
**Bacillus**	Butyrivibrio_2	0.8004	negative
**Bacillus**	Tyzzerella	0.6691	positive
**Bacillus**	Alkalibacterium	0.7903	positive
**Bacillus**	Jeotgalicoccus	0.7406	positive
**Bacillus**	[Eubacterium]_brachy_group	0.9017	positive
**Bacillus**	Veillonellaceae_UCG-001	0.6516	negative
**Bacillus**	[Anaerorhabdus]_furcosa_group	0.8004	negative

**Bacillus**	Staphylococcus	0.8161	positive
**Bacillus**	Stenotrophomonas	0.7186	positive
**Bacillus**	Lachnospiraceae_ND3007_group	0.6516	negative
**Bacillus**	Oxalobacter	0.6386	positive
**Bacillus**	Tyzzerella_4	0.8205	positive
**Bacillus**	Parvibacter	0.777	positive
**Bacillus**	Ruminococcus_2	0.8338	negative
**Bacillus**	Lachnospiraceae_NK3A20_group	0.8004	negative
**Bacillus**	Butyricimonas	0.8004	negative
**Bacillus**	Anaerovorax	0.6516	negative
**Bacillus**	Blautia	0.9017	positive
**Bacillus**	Ruminococcaceae_UCG-013	0.6924	negative
**Bacillus**	Ruminiclostridium_5	0.6516	positive
**Rikenellaceae_RC9_gut_group**	Ruminococcaceae_UCG-014	0.9152	positive
**Rikenellaceae_RC9_gut_group**	Lysinibacillus	0.6606	positive
**Rikenellaceae_RC9_gut_group**	Ruminococcaceae_UCG-005	0.9758	positive
**Rikenellaceae_RC9_gut_group**	Alistipes	0.9758	positive
**Rikenellaceae_RC9_gut_group**	dgA-11_gut_group	0.903	positive
**Rikenellaceae_RC9_gut_group**	Christensenellaceae_R-7_group	0.7818	positive
**Rikenellaceae_RC9_gut_group**	Prevotellaceae_UCG-004	0.9394	positive
**Rikenellaceae_RC9_gut_group**	Romboutsia	0.9394	positive
**Rikenellaceae_RC9_gut_group**	Clostridium_sensu_stricto_1	0.8303	positive

**Rikenellaceae_RC9_gut_group**	Ruminococcaceae_UCG-010	0.9394	positive
**Rikenellaceae_RC9_gut_group**	Treponema_2	0.9058	positive
**Rikenellaceae_RC9_gut_group**	Akkermansia	0.7455	positive
**Rikenellaceae_RC9_gut_group**	Ruminococcaceae_UCG-002	0.6727	positive
**Rikenellaceae_RC9_gut_group**	Succiniclasticum	0.7424	negative
**Rikenellaceae_RC9_gut_group**	Paeniclostridium	0.8788	positive
**Rikenellaceae_RC9_gut_group**	Mogibacterium	0.6688	negative
**Rikenellaceae_RC9_gut_group**	Ruminiclostridium_6	0.7576	positive
**Rikenellaceae_RC9_gut_group**	Erysipelotrichaceae_UCG-009	0.7564	negative
**Rikenellaceae_RC9_gut_group**	Turicibacter	0.9245	positive
**Rikenellaceae_RC9_gut_group**	Ruminococcaceae_UCG-009	0.8545	positive
**Rikenellaceae_RC9_gut_group**	Enterococcus	0.697	positive
**Rikenellaceae_RC9_gut_group**	Lachnospiraceae_XPB1014_group	0.6788	negative
**Rikenellaceae_RC9_gut_group**	Desulfovibrio	0.7176	negative
**Rikenellaceae_RC9_gut_group**	Aerococcus	0.8986	positive
**Rikenellaceae_RC9_gut_group**	Glutamicibacter	0.8469	positive
**Rikenellaceae_RC9_gut_group**	Butyrivibrio_2	0.7176	negative
**Rikenellaceae_RC9_gut_group**	Tyzzerella	0.8061	positive
**Rikenellaceae_RC9_gut_group**	Alkalibacterium	0.7128	positive
**Rikenellaceae_RC9_gut_group**	Jeotgalicoccus	0.7792	positive
**Rikenellaceae_RC9_gut_group**	[Eubacterium]_brachy_group	0.8262	positive
**Rikenellaceae_RC9_gut_group**	[Anaerorhabdus]_furcosa_group	0.6918	negative

**Rikenellaceae_RC9_gut_group**	Gemmobacter	0.6636	positive
**Rikenellaceae_RC9_gut_group**	Staphylococcus	0.7629	positive
**Rikenellaceae_RC9_gut_group**	Stenotrophomonas	0.7101	positive
**Rikenellaceae_RC9_gut_group**	Oxalobacter	0.6786	positive
**Rikenellaceae_RC9_gut_group**	Tyzzerella_4	0.7823	positive
**Rikenellaceae_RC9_gut_group**	Parvibacter	0.6935	positive
**Rikenellaceae_RC9_gut_group**	Ruminococcus_2	0.7564	negative
**Rikenellaceae_RC9_gut_group**	Lachnospiraceae_NK3A20_group	0.7176	negative
**Rikenellaceae_RC9_gut_group**	Butyricimonas	0.6918	negative
**Rikenellaceae_RC9_gut_group**	Blautia	0.8262	positive
**Rikenellaceae_RC9_gut_group**	Ruminiclostridium_5	0.7511	positive
**Ruminococcaceae_UCG-014**	Lysinibacillus	0.7333	positive
**Ruminococcaceae_UCG-014**	Ruminococcaceae_UCG-005	0.8909	positive
**Ruminococcaceae_UCG-014**	Alistipes	0.9273	positive
**Ruminococcaceae_UCG-014**	dgA-11_gut_group	0.9273	positive
**Ruminococcaceae_UCG-014**	Christensenellaceae_R-7_group	0.8061	positive
**Ruminococcaceae_UCG-014**	Prevotellaceae_UCG-004	0.9515	positive
**Ruminococcaceae_UCG-014**	Romboutsia	0.9152	positive
**Ruminococcaceae_UCG-014**	Clostridium_sensu_stricto_1	0.6606	positive
**Ruminococcaceae_UCG-014**	Ruminococcaceae_UCG-010	0.9515	positive
**Ruminococcaceae_UCG-014**	Treponema_2	0.8754	positive
**Ruminococcaceae_UCG-014**	Akkermansia	0.8182	positive

**Ruminococcaceae_UCG-014**	Ruminococcaceae_UCG-002	0.7091	positive
**Ruminococcaceae_UCG-014**	Succiniclasticum	0.7301	negative
**Ruminococcaceae_UCG-014**	Paeniclostridium	0.903	positive
**Ruminococcaceae_UCG-014**	Prevotellaceae_UCG-001	0.6727	positive
**Ruminococcaceae_UCG-014**	Ruminiclostridium_6	0.7455	positive
**Ruminococcaceae_UCG-014**	Erysipelotrichaceae_UCG-009	0.653	negative
**Ruminococcaceae_UCG-014**	Turicibacter	0.834	positive
**Ruminococcaceae_UCG-014**	Ruminococcaceae_UCG-009	0.8667	positive
**Ruminococcaceae_UCG-014**	Enterococcus	0.7818	positive
**Ruminococcaceae_UCG-014**	Aerococcus	0.7693	positive
**Ruminococcaceae_UCG-014**	Glutamicibacter	0.6918	positive
**Ruminococcaceae_UCG-014**	Oscillibacter	0.7091	positive
**Ruminococcaceae_UCG-014**	Tyzzerella	0.8061	positive
**Ruminococcaceae_UCG-014**	Alkalibacterium	0.6878	positive
**Ruminococcaceae_UCG-014**	[Eubacterium]_brachy_group	0.8876	positive
**Ruminococcaceae_UCG-014**	[Anaerorhabdus]_furcosa_group	0.64	negative
**Ruminococcaceae_UCG-014**	Staphylococcus	0.7128	positive
**Ruminococcaceae_UCG-014**	Tyzzerella_4	0.7306	positive
**Ruminococcaceae_UCG-014**	Parvibacter	0.7382	positive
**Ruminococcaceae_UCG-014**	Ruminococcus_2	0.653	negative
**Ruminococcaceae_UCG-014**	Butyricimonas	0.64	negative
**Ruminococcaceae_UCG-014**	Blautia	0.8876	positive

**Ruminococcaceae_UCG-014**	Ruminiclostridium_5	0.8262	positive
**Candidatus_Saccharimonas**	Prevotella_1	0.8788	positive
**Candidatus_Saccharimonas**	Prevotellaceae_UCG-003	0.8303	positive
**Candidatus_Saccharimonas**	Ruminococcaceae_NK4A214_group	0.8909	positive
**Candidatus_Saccharimonas**	Mogibacterium	0.8283	positive
**Candidatus_Saccharimonas**	Erysipelotrichaceae_UCG-009	0.7952	positive
**Candidatus_Saccharimonas**	Oribacterium	0.7504	positive
**Candidatus_Saccharimonas**	Lachnospiraceae_XPB1014_group	0.7306	positive
**Candidatus_Saccharimonas**	Desulfovibrio	0.7306	positive
**Candidatus_Saccharimonas**	Lachnospiraceae_AC2044_group	0.681	positive
**Candidatus_Saccharimonas**	Glutamicibacter	0.6788	negative
**Candidatus_Saccharimonas**	Butyrivibrio_2	0.8081	positive
**Candidatus_Saccharimonas**	Alkalibacterium	0.7754	negative
**Candidatus_Saccharimonas**	Ruminiclostridium_1	0.7455	positive
**Candidatus_Saccharimonas**	Jeotgalicoccus	0.6688	negative
**Candidatus_Saccharimonas**	[Anaerorhabdus]_furcosa_group	0.7823	positive
**Candidatus_Saccharimonas**	Staphylococcus	0.7504	negative
**Candidatus_Saccharimonas**	Lachnospiraceae_NK4A136_group	0.8129	positive
**Candidatus_Saccharimonas**	Lachnospiraceae_ND3007_group	0.8125	positive
**Candidatus_Saccharimonas**	Solobacterium	0.6691	positive
**Candidatus_Saccharimonas**	Ruminococcus_2	0.7952	positive
**Candidatus_Saccharimonas**	Lachnospiraceae_NK3A20_group	0.8081	positive

**Candidatus_Saccharimonas**	Butyricimonas	0.7823	positive
**Candidatus_Saccharimonas**	Corynebacterium_1	0.6442	negative
**Lysinibacillus**	Alistipes	0.6606	positive
**Lysinibacillus**	Prevotellaceae_UCG-004	0.6364	positive
**Lysinibacillus**	Prevotella_1	0.8667	negative
**Lysinibacillus**	Romboutsia	0.6848	positive
**Lysinibacillus**	Treponema_2	0.6991	positive
**Lysinibacillus**	Prevotellaceae_UCG-003	0.8545	negative
**Lysinibacillus**	Succiniclasticum	0.8896	negative
**Lysinibacillus**	Mogibacterium	0.816	negative
**Lysinibacillus**	Erysipelotrichaceae_UCG-009	0.8469	negative
**Lysinibacillus**	Turicibacter	0.7952	positive
**Lysinibacillus**	Oribacterium	0.8317	negative
**Lysinibacillus**	Lachnospiraceae_XPB1014_group	0.8857	negative
**Lysinibacillus**	Desulfovibrio	0.7952	negative
**Lysinibacillus**	Pseudobutyrivibrio	0.8128	negative
**Lysinibacillus**	Aerococcus	0.7693	positive
**Lysinibacillus**	Glutamicibacter	0.7176	positive
**Lysinibacillus**	Butyrivibrio_2	0.8599	negative
**Lysinibacillus**	Alkalibacterium	0.8066	positive
**Lysinibacillus**	[Eubacterium]_brachy_group	0.7442	positive
**Lysinibacillus**	Veillonellaceae_UCG-001	0.7989	negative

**Lysinibacillus**	[Anaerorhabdus]_furcosa_group	0.8986	negative
**Lysinibacillus**	Staphylococcus	0.8567	positive
**Lysinibacillus**	Lachnospiraceae_ND3007_group	0.8467	negative
**Lysinibacillus**	Solobacterium	0.7511	negative
**Lysinibacillus**	Ruminococcus_2	0.8469	negative
**Lysinibacillus**	Lachnospiraceae_NK3A20_group	0.8599	negative
**Lysinibacillus**	Butyricimonas	0.8986	negative
**Lysinibacillus**	Anaerovorax	0.7442	negative
**Lysinibacillus**	Blautia	0.7442	positive
**Lysinibacillus**	Corynebacterium_1	0.681	positive
**Ruminococcaceae_UCG-005**	Alistipes	0.9636	positive
**Ruminococcaceae_UCG-005**	dgA-11_gut_group	0.9152	positive
**Ruminococcaceae_UCG-005**	Christensenellaceae_R-7_group	0.8545	positive
**Ruminococcaceae_UCG-005**	Prevotellaceae_UCG-004	0.9394	positive
**Ruminococcaceae_UCG-005**	Romboutsia	0.9394	positive
**Ruminococcaceae_UCG-005**	Clostridium_sensu_stricto_1	0.8061	positive
**Ruminococcaceae_UCG-005**	Ruminococcaceae_UCG-010	0.9394	positive
**Ruminococcaceae_UCG-005**	Treponema_2	0.8997	positive
**Ruminococcaceae_UCG-005**	Akkermansia	0.7576	positive
**Ruminococcaceae_UCG-005**	Ruminococcaceae_UCG-002	0.7697	positive
**Ruminococcaceae_UCG-005**	Succiniclasticum	0.6565	negative
**Ruminococcaceae_UCG-005**	Paeniclostridium	0.9152	positive

**Ruminococcaceae_UCG-005**	Prevotellaceae_UCG-001	0.6727	positive
**Ruminococcaceae_UCG-005**	Ruminiclostridium_6	0.8182	positive
**Ruminococcaceae_UCG-005**	Turicibacter	0.8857	positive
**Ruminococcaceae_UCG-005**	Ruminococcaceae_UCG-009	0.9273	positive
**Ruminococcaceae_UCG-005**	Enterococcus	0.7697	positive
**Ruminococcaceae_UCG-005**	Desulfovibrio	0.64	negative
**Ruminococcaceae_UCG-005**	Aerococcus	0.8599	positive
**Ruminococcaceae_UCG-005**	Glutamicibacter	0.7952	positive
**Ruminococcaceae_UCG-005**	Oscillibacter	0.6606	positive
**Ruminococcaceae_UCG-005**	Tyzzerella	0.8424	positive
**Ruminococcaceae_UCG-005**	Jeotgalicoccus	0.7178	positive
**Ruminococcaceae_UCG-005**	[Eubacterium]_brachy_group	0.7784	positive
**Ruminococcaceae_UCG-005**	Gemmobacter	0.6636	positive
**Ruminococcaceae_UCG-005**	Staphylococcus	0.6628	positive
**Ruminococcaceae_UCG-005**	Stenotrophomonas	0.6486	positive
**Ruminococcaceae_UCG-005**	Oxalobacter	0.6786	positive
**Ruminococcaceae_UCG-005**	Tyzzerella_4	0.7823	positive
**Ruminococcaceae_UCG-005**	Parvibacter	0.6338	positive
**Ruminococcaceae_UCG-005**	Blautia	0.7784	positive
**Ruminococcaceae_UCG-005**	Ruminiclostridium_5	0.7989	positive
**Alistipes**	dgA-11_gut_group	0.8667	positive
**Alistipes**	Christensenellaceae_R-7_group	0.7818	positive

**Alistipes**	Prevotellaceae_UCG-004	0.9758	positive
**Alistipes**	Romboutsia	0.903	positive
**Alistipes**	Clostridium_sensu_stricto_1	0.8545	positive
**Alistipes**	Ruminococcaceae_UCG-010	0.9394	positive
**Alistipes**	Treponema_2	0.9362	positive
**Alistipes**	Akkermansia	0.8545	positive
**Alistipes**	Ruminococcaceae_UCG-002	0.6848	positive
**Alistipes**	Succiniclasticum	0.7424	negative
**Alistipes**	Paeniclostridium	0.9152	positive
**Alistipes**	Mogibacterium	0.6319	negative
**Alistipes**	Ruminiclostridium_6	0.7697	positive
**Alistipes**	Erysipelotrichaceae_UCG-009	0.7176	negative
**Alistipes**	Turicibacter	0.9116	positive
**Alistipes**	Oribacterium	0.6441	negative
**Alistipes**	Ruminococcaceae_UCG-009	0.8545	positive
**Alistipes**	Enterococcus	0.7455	positive
**Alistipes**	Lachnospiraceae_XPB1014_group	0.6918	negative
**Alistipes**	Desulfovibrio	0.7306	negative
**Alistipes**	Aerococcus	0.8728	positive
**Alistipes**	Glutamicibacter	0.8211	positive
**Alistipes**	Butyrivibrio_2	0.6918	negative
**Alistipes**	Oscillibacter	0.6606	positive

**Alistipes**	Tyzzerella	0.7818	positive
**Alistipes**	Alkalibacterium	0.7191	positive
**Alistipes**	Jeotgalicoccus	0.7056	positive
**Alistipes**	[Eubacterium]_brachy_group	0.874	positive
**Alistipes**	[Anaerorhabdus]_furcosa_group	0.6788	negative
**Alistipes**	Staphylococcus	0.7441	positive
**Alistipes**	Stenotrophomonas	0.6964	positive
**Alistipes**	Tyzzerella_4	0.7435	positive
**Alistipes**	Parvibacter	0.7531	positive
**Alistipes**	Ruminococcus_2	0.7176	negative
**Alistipes**	Lachnospiraceae_NK3A20_group	0.6918	negative
**Alistipes**	Butyricimonas	0.6788	negative
**Alistipes**	Blautia	0.874	positive
**Alistipes**	Ruminiclostridium_5	0.7511	positive
**dgA-11_gut_group**	Christensenellaceae_R-7_group	0.9152	positive
**dgA-11_gut_group**	Prevotellaceae_UCG-004	0.903	positive
**dgA-11_gut_group**	Romboutsia	0.903	positive
**dgA-11_gut_group**	Ruminococcaceae_UCG-010	0.9636	positive
**dgA-11_gut_group**	Treponema_2	0.8146	positive
**dgA-11_gut_group**	Akkermansia	0.6606	positive
**dgA-11_gut_group**	Ruminococcaceae_UCG-002	0.8303	positive
**dgA-11_gut_group**	Paeniclostridium	0.8545	positive

**dgA-11_gut_group**	Prevotellaceae_UCG-001	0.7818	positive
**dgA-11_gut_group**	Ruminiclostridium_6	0.8545	positive
**dgA-11_gut_group**	Turicibacter	0.7306	positive
**dgA-11_gut_group**	Ruminococcaceae_UCG-009	0.9394	positive
**dgA-11_gut_group**	Enterococcus	0.8061	positive
**dgA-11_gut_group**	Family_XIII_AD3011_group	0.6869	positive
**dgA-11_gut_group**	Aerococcus	0.6788	positive
**dgA-11_gut_group**	Oscillibacter	0.7455	positive
**dgA-11_gut_group**	Tyzzerella	0.9152	positive
**dgA-11_gut_group**	[Eubacterium]_brachy_group	0.7306	positive
**dgA-11_gut_group**	Tyzzerella_4	0.6918	positive
**dgA-11_gut_group**	Blautia	0.7306	positive
**dgA-11_gut_group**	Ruminiclostridium_5	0.8467	positive
**Christensenellaceae_R-7_group**	Prevotellaceae_UCG-004	0.8545	positive
**Christensenellaceae_R-7_group**	Romboutsia	0.7697	positive
**Christensenellaceae_R-7_group**	Ruminococcaceae_UCG-010	0.903	positive
**Christensenellaceae_R-7_group**	Treponema_2	0.693	positive
**Christensenellaceae_R-7_group**	Ruminococcaceae_UCG-002	0.9394	positive
**Christensenellaceae_R-7_group**	Paeniclostridium	0.8303	positive
**Christensenellaceae_R-7_group**	Prevotellaceae_UCG-001	0.9394	positive
**Christensenellaceae_R-7_group**	Ruminiclostridium_6	0.8182	positive
**Christensenellaceae_R-7_group**	Terrisporobacter	0.7333	positive

**Christensenellaceae_R-7_group**	Ruminococcaceae_UCG-009	0.9636	positive
**Christensenellaceae_R-7_group**	Enterococcus	0.8667	positive
**Christensenellaceae_R-7_group**	Family_XIII_AD3011_group	0.8328	positive
**Christensenellaceae_R-7_group**	Oscillibacter	0.7455	positive
**Christensenellaceae_R-7_group**	Tyzzerella	0.9636	positive
**Christensenellaceae_R-7_group**	Ruminiclostridium_1	0.7697	positive
**Christensenellaceae_R-7_group**	Tyzzerella_4	0.6918	positive
**Christensenellaceae_R-7_group**	Ruminiclostridium_5	0.833	positive
**Prevotellaceae_UCG-004**	Romboutsia	0.8667	positive
**Prevotellaceae_UCG-004**	Clostridium_sensu_stricto_1	0.8061	positive
**Prevotellaceae_UCG-004**	Ruminococcaceae_UCG-010	0.9758	positive
**Prevotellaceae_UCG-004**	Treponema_2	0.9362	positive
**Prevotellaceae_UCG-004**	Akkermansia	0.8424	positive
**Prevotellaceae_UCG-004**	Ruminococcaceae_UCG-002	0.7333	positive
**Prevotellaceae_UCG-004**	Succiniclasticum	0.681	negative
**Prevotellaceae_UCG-004**	Paeniclostridium	0.9273	positive
**Prevotellaceae_UCG-004**	Prevotellaceae_UCG-001	0.7212	positive
**Prevotellaceae_UCG-004**	Ruminiclostridium_6	0.8182	positive
**Prevotellaceae_UCG-004**	Turicibacter	0.834	positive
**Prevotellaceae_UCG-004**	Ruminococcaceae_UCG-009	0.8909	positive
**Prevotellaceae_UCG-004**	Enterococcus	0.8061	positive
**Prevotellaceae_UCG-004**	Desulfovibrio	0.64	negative

**Prevotellaceae_UCG-004**	Aerococcus	0.7693	positive
**Prevotellaceae_UCG-004**	Glutamicibacter	0.6918	positive
**Prevotellaceae_UCG-004**	Oscillibacter	0.6848	positive
**Prevotellaceae_UCG-004**	Tyzzerella	0.8303	positive
**Prevotellaceae_UCG-004**	[Eubacterium]_brachy_group	0.8876	positive
**Prevotellaceae_UCG-004**	Staphylococcus	0.6503	positive
**Prevotellaceae_UCG-004**	Tyzzerella_4	0.7306	positive
**Prevotellaceae_UCG-004**	Parvibacter	0.7382	positive
**Prevotellaceae_UCG-004**	Blautia	0.8876	positive
**Prevotellaceae_UCG-004**	Ruminiclostridium_5	0.8262	positive
**Prevotella_1**	Prevotellaceae_UCG-003	0.9879	positive
**Prevotella_1**	Ruminococcaceae_NK4A214_group	0.7455	positive
**Prevotella_1**	Succiniclasticum	0.8037	positive
**Prevotella_1**	Mogibacterium	0.8774	positive
**Prevotella_1**	Erysipelotrichaceae_UCG-009	0.8986	positive
**Prevotella_1**	Turicibacter	0.6918	negative
**Prevotella_1**	Oribacterium	0.9192	positive
**Prevotella_1**	Lachnospiraceae_XPB1014_group	0.9245	positive
**Prevotella_1**	Desulfovibrio	0.8857	positive
**Prevotella_1**	Pseudobutyrivibrio	0.768	positive
**Prevotella_1**	Aerococcus	0.7176	negative
**Prevotella_1**	Glutamicibacter	0.7693	negative

**Prevotella_1**	Butyrivibrio_2	0.9245	positive
**Prevotella_1**	Alkalibacterium	0.8817	negative
**Prevotella_1**	Jeotgalicoccus	0.6565	negative
**Prevotella_1**	Veillonellaceae_UCG-001	0.8398	positive
**Prevotella_1**	[Anaerorhabdus]_furcosa_group	0.9374	positive
**Prevotella_1**	Staphylococcus	0.8317	negative
**Prevotella_1**	Stenotrophomonas	0.6555	negative
**Prevotella_1**	Methanobrevibacter	0.6626	positive
**Prevotella_1**	Lachnospiraceae_ND3007_group	0.8876	positive
**Prevotella_1**	Solobacterium	0.792	positive
**Prevotella_1**	Ruminococcus_2	0.8986	positive
**Prevotella_1**	Lachnospiraceae_NK3A20_group	0.9245	positive
**Prevotella_1**	Butyricimonas	0.9374	positive
**Prevotella_1**	Anaerovorax	0.8125	positive
**Prevotella_1**	Corynebacterium_1	0.7547	negative
**Romboutsia**	Clostridium_sensu_stricto_1	0.6727	positive
**Romboutsia**	Ruminococcaceae_UCG-010	0.8909	positive
**Romboutsia**	Treponema_2	0.8146	positive
**Romboutsia**	Akkermansia	0.7455	positive
**Romboutsia**	Ruminococcaceae_UCG-002	0.7212	positive
**Romboutsia**	Succiniclasticum	0.7056	negative
**Romboutsia**	Paeniclostridium	0.9152	positive

**Romboutsia**	Mogibacterium	0.6688	negative
**Romboutsia**	Ruminiclostridium_6	0.6848	positive
**Romboutsia**	Erysipelotrichaceae_UCG-009	0.653	negative
**Romboutsia**	Turicibacter	0.8986	positive
**Romboutsia**	Ruminococcaceae_UCG-009	0.8667	positive
**Romboutsia**	Enterococcus	0.7697	positive
**Romboutsia**	Aerococcus	0.8857	positive
**Romboutsia**	Glutamicibacter	0.8211	positive
**Romboutsia**	Tyzzerella	0.7697	positive
**Romboutsia**	Alkalibacterium	0.6753	positive
**Romboutsia**	Jeotgalicoccus	0.681	positive
**Romboutsia**	[Eubacterium]_brachy_group	0.7511	positive
**Romboutsia**	[Anaerorhabdus]_furcosa_group	0.64	negative
**Romboutsia**	Staphylococcus	0.7754	positive
**Romboutsia**	Oxalobacter	0.7531	positive
**Romboutsia**	Tyzzerella_4	0.7693	positive
**Romboutsia**	Ruminococcus_2	0.653	negative
**Romboutsia**	Butyricimonas	0.64	negative
**Romboutsia**	Blautia	0.7511	positive
**Romboutsia**	Ruminiclostridium_5	0.7852	positive
**Clostridium_sensu_stricto_1**	Ruminococcaceae_UCG-010	0.7576	positive
**Clostridium_sensu_stricto_1**	Treponema_2	0.8268	positive

**Clostridium_sensu_stricto_1**	Akkermansia	0.6606	positive
**Clostridium_sensu_stricto_1**	Succiniclasticum	0.7056	negative
**Clostridium_sensu_stricto_1**	Paeniclostridium	0.7333	positive
**Clostridium_sensu_stricto_1**	Erysipelotrichaceae_UCG-009	0.6918	negative
**Clostridium_sensu_stricto_1**	Turicibacter	0.834	positive
**Clostridium_sensu_stricto_1**	Oribacterium	0.6566	negative
**Clostridium_sensu_stricto_1**	Desulfovibrio	0.7564	negative
**Clostridium_sensu_stricto_1**	Aerococcus	0.7952	positive
**Clostridium_sensu_stricto_1**	Glutamicibacter	0.7176	positive
**Clostridium_sensu_stricto_1**	Butyrivibrio_2	0.6659	negative
**Clostridium_sensu_stricto_1**	Jeotgalicoccus	0.7915	positive
**Clostridium_sensu_stricto_1**	[Eubacterium]_brachy_group	0.7989	positive
**Clostridium_sensu_stricto_1**	Staphylococcus	0.6691	positive
**Clostridium_sensu_stricto_1**	Oxalobacter	0.6338	positive
**Clostridium_sensu_stricto_1**	Tyzzerella_4	0.6918	positive
**Clostridium_sensu_stricto_1**	Ruminococcus_2	0.6918	negative
**Clostridium_sensu_stricto_1**	Lachnospiraceae_NK3A20_group	0.6659	negative
**Clostridium_sensu_stricto_1**	Blautia	0.7989	positive
**Clostridium_sensu_stricto_1**	Ruminococcaceae_UCG-013	0.7531	negative
**Clostridium_sensu_stricto_1**	Corynebacterium_1	0.7178	positive
**Clostridium_sensu_stricto_1**	Ruminiclostridium_5	0.6418	positive
**Ruminococcaceae_UCG-010**	Treponema_2	0.8936	positive

**Ruminococcaceae_UCG-010**	Akkermansia	0.7576	positive
**Ruminococcaceae_UCG-010**	Ruminococcaceae_UCG-002	0.7818	positive
**Ruminococcaceae_UCG-010**	Paeniclostridium	0.9152	positive
**Ruminococcaceae_UCG-010**	Prevotellaceae_UCG-001	0.7697	positive
**Ruminococcaceae_UCG-010**	Ruminiclostridium_6	0.8303	positive
**Ruminococcaceae_UCG-010**	Turicibacter	0.7952	positive
**Ruminococcaceae_UCG-010**	Ruminococcaceae_UCG-009	0.9152	positive
**Ruminococcaceae_UCG-010**	Enterococcus	0.8303	positive
**Ruminococcaceae_UCG-010**	Family_XIII_AD3011_group	0.6322	positive
**Ruminococcaceae_UCG-010**	Aerococcus	0.7306	positive
**Ruminococcaceae_UCG-010**	Glutamicibacter	0.64	positive
**Ruminococcaceae_UCG-010**	Oscillibacter	0.6848	positive
**Ruminococcaceae_UCG-010**	Tyzzerella	0.8909	positive
**Ruminococcaceae_UCG-010**	[Eubacterium]_brachy_group	0.8398	positive
**Ruminococcaceae_UCG-010**	Tyzzerella_4	0.7306	positive
**Ruminococcaceae_UCG-010**	Parvibacter	0.6786	positive
**Ruminococcaceae_UCG-010**	Blautia	0.8398	positive
**Ruminococcaceae_UCG-010**	Ruminiclostridium_5	0.874	positive
**Treponema_2**	Akkermansia	0.8207	positive
**Treponema_2**	Succiniclasticum	0.8	negative
**Treponema_2**	Paeniclostridium	0.8511	positive
**Treponema_2**	Mogibacterium	0.6523	negative

**Treponema_2**	Ruminiclostridium_6	0.8268	positive
**Treponema_2**	Erysipelotrichaceae_UCG-009	0.7328	negative
**Treponema_2**	Turicibacter	0.8884	positive
**Treponema_2**	Oribacterium	0.7464	negative
**Treponema_2**	Ruminococcaceae_UCG-009	0.7842	positive
**Treponema_2**	Enterococcus	0.6505	positive
**Treponema_2**	Lachnospiraceae_XPB1014_group	0.7587	negative
**Treponema_2**	Desulfovibrio	0.8041	negative
**Treponema_2**	Aerococcus	0.8365	positive
**Treponema_2**	Glutamicibacter	0.7717	positive
**Treponema_2**	Butyrivibrio_2	0.7522	negative
**Treponema_2**	Tyzzerella	0.6444	positive
**Treponema_2**	Alkalibacterium	0.6993	positive
**Treponema_2**	Jeotgalicoccus	0.7323	positive
**Treponema_2**	[Eubacterium]_brachy_group	0.8903	positive
**Treponema_2**	[Anaerorhabdus]_furcosa_group	0.7393	negative
**Treponema_2**	Staphylococcus	0.7244	positive
**Treponema_2**	Stenotrophomonas	0.6575	positive
**Treponema_2**	Tyzzerella_4	0.642	positive
**Treponema_2**	Parvibacter	0.7405	positive
**Treponema_2**	Ruminococcus_2	0.7328	negative
**Treponema_2**	Lachnospiraceae_NK3A20_group	0.7522	negative

**Treponema_2**	Butyricimonas	0.7393	negative
**Treponema_2**	Anaerovorax	0.6643	negative
**Treponema_2**	Blautia	0.8903	positive
**Treponema_2**	Corynebacterium_1	0.6954	positive
**Treponema_2**	Ruminiclostridium_5	0.7807	positive
**Akkermansia**	Succiniclasticum	0.6442	negative
**Akkermansia**	Paeniclostridium	0.8667	positive
**Akkermansia**	Ruminiclostridium_6	0.6485	positive
**Akkermansia**	Turicibacter	0.7564	positive
**Akkermansia**	Ruminococcaceae_UCG-009	0.6848	positive
**Akkermansia**	Enterococcus	0.7697	positive
**Akkermansia**	Aerococcus	0.7176	positive
**Akkermansia**	Glutamicibacter	0.6918	positive
**Akkermansia**	Oscillibacter	0.6848	positive
**Akkermansia**	Alkalibacterium	0.7066	positive
**Akkermansia**	[Eubacterium]_brachy_group	0.792	positive
**Akkermansia**	Staphylococcus	0.6816	positive
**Akkermansia**	Parvibacter	0.7233	positive
**Akkermansia**	Blautia	0.792	positive
**Prevotellaceae_UCG-003**	Ruminococcaceae_NK4A214_group	0.697	positive
**Prevotellaceae_UCG-003**	Succiniclasticum	0.8037	positive
**Prevotellaceae_UCG-003**	Mogibacterium	0.8406	positive

**Prevotellaceae_UCG-003**	Erysipelotrichaceae_UCG-009	0.8986	positive
**Prevotellaceae_UCG-003**	Turicibacter	0.6788	negative
**Prevotellaceae_UCG-003**	Oribacterium	0.9192	positive
**Prevotellaceae_UCG-003**	Lachnospiraceae_XPB1014_group	0.9245	positive
**Prevotellaceae_UCG-003**	Desulfovibrio	0.8857	positive
**Prevotellaceae_UCG-003**	Pseudobutyrivibrio	0.768	positive
**Prevotellaceae_UCG-003**	Aerococcus	0.6918	negative
**Prevotellaceae_UCG-003**	Glutamicibacter	0.7435	negative
**Prevotellaceae_UCG-003**	Butyrivibrio_2	0.9245	positive
**Prevotellaceae_UCG-003**	Alkalibacterium	0.8567	negative
**Prevotellaceae_UCG-003**	Jeotgalicoccus	0.6442	negative
**Prevotellaceae_UCG-003**	Veillonellaceae_UCG-001	0.8398	positive
**Prevotellaceae_UCG-003**	[Anaerorhabdus]_furcosa_group	0.9374	positive
**Prevotellaceae_UCG-003**	Staphylococcus	0.7816	negative
**Prevotellaceae_UCG-003**	Stenotrophomonas	0.7033	negative
**Prevotellaceae_UCG-003**	Methanobrevibacter	0.7295	positive
**Prevotellaceae_UCG-003**	Lachnospiraceae_ND3007_group	0.8876	positive
**Prevotellaceae_UCG-003**	Solobacterium	0.792	positive
**Prevotellaceae_UCG-003**	Ruminococcus_2	0.8986	positive
**Prevotellaceae_UCG-003**	Lachnospiraceae_NK3A20_group	0.9245	positive
**Prevotellaceae_UCG-003**	Butyricimonas	0.9374	positive
**Prevotellaceae_UCG-003**	Anaerovorax	0.8125	positive

**Prevotellaceae_UCG-003**	Corynebacterium_1	0.7424	negative
**Ruminococcaceae_UCG-002**	Paeniclostridium	0.7697	positive
**Ruminococcaceae_UCG-002**	Prevotellaceae_UCG-001	0.9394	positive
**Ruminococcaceae_UCG-002**	Ruminiclostridium_6	0.7333	positive
**Ruminococcaceae_UCG-002**	Ruminococcaceae_UCG-009	0.9152	positive
**Ruminococcaceae_UCG-002**	Enterococcus	0.8788	positive
**Ruminococcaceae_UCG-002**	Family_XIII_AD3011_group	0.8024	positive
**Ruminococcaceae_UCG-002**	Oscillibacter	0.8545	positive
**Ruminococcaceae_UCG-002**	Tyzzerella	0.9152	positive
**Ruminococcaceae_UCG-002**	Ruminiclostridium_1	0.7697	positive
**Ruminococcaceae_UCG-002**	Ruminiclostridium_5	0.6896	positive
**Ruminococcaceae_NK4A214_group**	Mogibacterium	0.6565	positive
**Ruminococcaceae_NK4A214_group**	Erysipelotrichaceae_UCG-009	0.64	positive
**Ruminococcaceae_NK4A214_group**	Oribacterium	0.7003	positive
**Ruminococcaceae_NK4A214_group**	Desulfovibrio	0.6918	positive
**Ruminococcaceae_NK4A214_group**	Lachnospiraceae_AC2044_group	0.6688	positive
**Ruminococcaceae_NK4A214_group**	Butyrivibrio_2	0.6659	positive
**Ruminococcaceae_NK4A214_group**	Alkalibacterium	0.6378	negative
**Ruminococcaceae_NK4A214_group**	Ruminiclostridium_1	0.6727	positive
**Ruminococcaceae_NK4A214_group**	Lachnospiraceae_NK4A136_group	0.6503	positive
**Ruminococcaceae_NK4A214_group**	Ruminococcus_2	0.64	positive
**Ruminococcaceae_NK4A214_group**	Lachnospiraceae_NK3A20_group	0.6659	positive

**Ruminococcaceae_NK4A214_group**	Corynebacterium_1	0.681	negative
**Succiniclasticum**	Mogibacterium	0.8882	positive
**Succiniclasticum**	Erysipelotrichaceae_UCG-009	0.9097	positive
**Succiniclasticum**	Turicibacter	0.9097	negative
**Succiniclasticum**	Oribacterium	0.8356	positive
**Succiniclasticum**	Lachnospiraceae_XPB1014_group	0.9359	positive
**Succiniclasticum**	Desulfovibrio	0.8967	positive
**Succiniclasticum**	Pseudobutyrivibrio	0.7775	positive
**Succiniclasticum**	Aerococcus	0.8705	negative
**Succiniclasticum**	Glutamicibacter	0.805	negative
**Succiniclasticum**	Butyrivibrio_2	0.9359	positive
**Succiniclasticum**	Alkalibacterium	0.8419	negative
**Succiniclasticum**	Jeotgalicoccus	0.7888	negative
**Succiniclasticum**	[Eubacterium]_brachy_group	0.8709	negative
**Succiniclasticum**	Veillonellaceae_UCG-001	0.8502	positive
**Succiniclasticum**	[Anaerorhabdus]_furcosa_group	0.949	positive
**Succiniclasticum**	Staphylococcus	0.8926	negative
**Succiniclasticum**	Lachnospiraceae_ND3007_group	0.8986	positive
**Succiniclasticum**	Tyzzerella_4	0.661	negative
**Succiniclasticum**	Solobacterium	0.6567	positive
**Succiniclasticum**	Parvibacter	0.6718	negative
**Succiniclasticum**	Ruminococcus_2	0.9097	positive

**Succiniclasticum**	Lachnospiraceae_NK3A20_group	0.9359	positive
**Succiniclasticum**	Butyricimonas	0.949	positive
**Succiniclasticum**	Anaerovorax	0.8226	positive
**Succiniclasticum**	Blautia	0.8709	negative
**Succiniclasticum**	Corynebacterium_1	0.7391	negative
**Parabacteroides**	Ruminiclostridium_6	0.6727	negative
**Paeniclostridium**	Prevotellaceae_UCG-001	0.697	positive
**Paeniclostridium**	Ruminiclostridium_6	0.7212	positive
**Paeniclostridium**	Turicibacter	0.8211	positive
**Paeniclostridium**	Ruminococcaceae_UCG-009	0.8788	positive
**Paeniclostridium**	Enterococcus	0.9152	positive
**Paeniclostridium**	Aerococcus	0.7823	positive
**Paeniclostridium**	Glutamicibacter	0.6918	positive
**Paeniclostridium**	Tyzzerella	0.7576	positive
**Paeniclostridium**	[Eubacterium]_brachy_group	0.7852	positive
**Paeniclostridium**	Staphylococcus	0.6816	positive
**Paeniclostridium**	Tyzzerella_4	0.7047	positive
**Paeniclostridium**	Blautia	0.7852	positive
**Paeniclostridium**	Ruminiclostridium_5	0.8467	positive
**Mogibacterium**	Erysipelotrichaceae_UCG-009	0.9097	positive
**Mogibacterium**	Turicibacter	0.8705	negative
**Mogibacterium**	Oribacterium	0.8356	positive

**Mogibacterium**	Lachnospiraceae_XPB1014_group	0.9359	positive
**Mogibacterium**	Desulfovibrio	0.8967	positive
**Mogibacterium**	Pseudobutyrivibrio	0.7775	positive
**Mogibacterium**	Aerococcus	0.9097	negative
**Mogibacterium**	Glutamicibacter	0.9228	negative
**Mogibacterium**	Butyrivibrio_2	0.9359	positive
**Mogibacterium**	Alkalibacterium	0.8799	negative
**Mogibacterium**	Jeotgalicoccus	0.8634	negative
**Mogibacterium**	Veillonellaceae_UCG-001	0.8502	positive
**Mogibacterium**	[Anaerorhabdus]_furcosa_group	0.949	positive
**Mogibacterium**	Staphylococcus	0.9305	negative
**Mogibacterium**	Lachnospiraceae_NK4A136_group	0.7913	positive
**Mogibacterium**	Stenotrophomonas	0.6981	negative
**Mogibacterium**	Lachnospiraceae_ND3007_group	0.8986	positive
**Mogibacterium**	Oxalobacter	0.7926	negative
**Mogibacterium**	Solobacterium	0.6567	positive
**Mogibacterium**	Ruminococcus_2	0.9097	positive
**Mogibacterium**	Lachnospiraceae_NK3A20_group	0.9359	positive
**Mogibacterium**	Butyricimonas	0.949	positive
**Mogibacterium**	Anaerovorax	0.8226	positive
**Prevotellaceae_UCG-001**	Ruminiclostridium_6	0.6848	positive
**Prevotellaceae_UCG-001**	Ruminococcaceae_UCG-009	0.8424	positive

**Prevotellaceae_UCG-001**	Enterococcus	0.8424	positive
**Prevotellaceae_UCG-001**	Family_XIII_AD3011_group	0.8268	positive
**Prevotellaceae_UCG-001**	Lachnospiraceae_AC2044_group	0.7178	positive
**Prevotellaceae_UCG-001**	Oscillibacter	0.7697	positive
**Prevotellaceae_UCG-001**	Tyzzerella	0.9152	positive
**Prevotellaceae_UCG-001**	Ruminiclostridium_1	0.8182	positive
**Prevotellaceae_UCG-001**	Ruminiclostridium_5	0.6555	positive
**Ruminiclostridium_6**	Ruminococcaceae_UCG-009	0.8545	positive
**Ruminiclostridium_6**	Enterococcus	0.7091	positive
**Ruminiclostridium_6**	Oscillibacter	0.7333	positive
**Ruminiclostridium_6**	Tyzzerella	0.7455	positive
**Ruminiclostridium_6**	Ruminiclostridium_5	0.6828	positive
**Erysipelotrichaceae_UCG-009**	Turicibacter	0.8621	negative
**Erysipelotrichaceae_UCG-009**	Oribacterium	0.9005	positive
**Erysipelotrichaceae_UCG-009**	Lachnospiraceae_XPB1014_group	0.9172	positive
**Erysipelotrichaceae_UCG-009**	Desulfovibrio	0.931	positive
**Erysipelotrichaceae_UCG-009**	Pseudobutyrivibrio	0.6602	positive
**Erysipelotrichaceae_UCG-009**	Aerococcus	0.8621	negative
**Erysipelotrichaceae_UCG-009**	Glutamicibacter	0.8621	negative
**Erysipelotrichaceae_UCG-009**	Butyrivibrio_2	0.9862	positive
**Erysipelotrichaceae_UCG-009**	Alkalibacterium	0.9005	negative
**Erysipelotrichaceae_UCG-009**	Jeotgalicoccus	0.8574	negative

**Erysipelotrichaceae_UCG-009**	[Eubacterium]_brachy_group	0.7284	negative
**Erysipelotrichaceae_UCG-009**	Veillonellaceae_UCG-001	0.7502	positive
**Erysipelotrichaceae_UCG-009**	[Anaerorhabdus]_furcosa_group	0.9586	positive
**Erysipelotrichaceae_UCG-009**	Staphylococcus	0.9005	negative
**Erysipelotrichaceae_UCG-009**	Stenotrophomonas	0.7284	negative
**Erysipelotrichaceae_UCG-009**	Methanobrevibacter	0.7004	positive
**Erysipelotrichaceae_UCG-009**	Lachnospiraceae_ND3007_group	0.9032	positive
**Erysipelotrichaceae_UCG-009**	Ruminococcus_2	1.0	positive
**Erysipelotrichaceae_UCG-009**	Lachnospiraceae_NK3A20_group	0.9862	positive
**Erysipelotrichaceae_UCG-009**	Butyricimonas	0.9586	positive
**Erysipelotrichaceae_UCG-009**	Anaerovorax	0.7356	positive
**Erysipelotrichaceae_UCG-009**	Blautia	0.7284	negative
**Erysipelotrichaceae_UCG-009**	Ruminococcaceae_UCG-013	0.7398	positive
**Erysipelotrichaceae_UCG-009**	Corynebacterium_1	0.805	negative
**Terrisporobacter**	Ruminococcaceae_UCG-009	0.6485	positive
**Terrisporobacter**	Enterococcus	0.6485	positive
**Terrisporobacter**	Family_XIII_AD3011_group	0.7842	positive
**Terrisporobacter**	Ruminiclostridium_1	0.6848	positive
**Terrisporobacter**	Ruminiclostridium_5	0.7306	positive
**Turicibacter**	Oribacterium	0.7671	negative
**Turicibacter**	Ruminococcaceae_UCG-009	0.6918	positive
**Turicibacter**	Lachnospiraceae_XPB1014_group	0.8621	negative

**Turicibacter**	Desulfovibrio	0.8621	negative
**Turicibacter**	Aerococcus	0.9862	positive
**Turicibacter**	Glutamicibacter	0.931	positive
**Turicibacter**	Butyrivibrio_2	0.8621	negative
**Turicibacter**	Alkalibacterium	0.8205	positive
**Turicibacter**	Jeotgalicoccus	0.8705	positive
**Turicibacter**	[Eubacterium]_brachy_group	0.8522	positive
**Turicibacter**	Veillonellaceae_UCG-001	0.7284	negative
**Turicibacter**	[Anaerorhabdus]_furcosa_group	0.8621	negative
**Turicibacter**	Staphylococcus	0.9005	positive
**Turicibacter**	Stenotrophomonas	0.7065	positive
**Turicibacter**	Lachnospiraceae_ND3007_group	0.7284	negative
**Turicibacter**	Oxalobacter	0.8034	positive
**Turicibacter**	Tyzzerella_4	0.7655	positive
**Turicibacter**	Parvibacter	0.6602	positive
**Turicibacter**	Ruminococcus_2	0.8621	negative
**Turicibacter**	Lachnospiraceae_NK3A20_group	0.8621	negative
**Turicibacter**	Butyricimonas	0.8621	negative
**Turicibacter**	Anaerovorax	0.7284	negative
**Turicibacter**	Blautia	0.8522	positive
**Turicibacter**	Corynebacterium_1	0.6349	positive
**Turicibacter**	Ruminiclostridium_5	0.6847	positive

**Oribacterium**	Lachnospiraceae_XPB1014_group	0.9272	positive
**Oribacterium**	Desulfovibrio	0.9672	positive
**Oribacterium**	Aerococcus	0.7671	negative
**Oribacterium**	Glutamicibacter	0.7671	negative
**Oribacterium**	Butyrivibrio_2	0.9272	positive
**Oribacterium**	Alkalibacterium	0.7742	negative
**Oribacterium**	Jeotgalicoccus	0.7596	negative
**Oribacterium**	[Eubacterium]_brachy_group	0.6516	negative
**Oribacterium**	Veillonellaceae_UCG-001	0.8383	positive
**Oribacterium**	[Anaerorhabdus]_furcosa_group	0.9138	positive
**Oribacterium**	Staphylococcus	0.7742	negative
**Oribacterium**	Lachnospiraceae_ND3007_group	0.789	positive
**Oribacterium**	Solobacterium	0.6904	positive
**Oribacterium**	Ruminococcus_2	0.9005	positive
**Oribacterium**	Lachnospiraceae_NK3A20_group	0.9272	positive
**Oribacterium**	Butyricimonas	0.9138	positive
**Oribacterium**	Anaerovorax	0.8665	positive
**Oribacterium**	Blautia	0.6516	negative
**Oribacterium**	Ruminococcaceae_UCG-013	0.6847	positive
**Oribacterium**	Corynebacterium_1	0.8419	negative
**Ruminococcaceae_UCG-009**	Enterococcus	0.8424	positive
**Ruminococcaceae_UCG-009**	Family_XIII_AD3011_group	0.7173	positive

**Ruminococcaceae_UCG-009**	Aerococcus	0.64	positive
**Ruminococcaceae_UCG-009**	Oscillibacter	0.7818	positive
**Ruminococcaceae_UCG-009**	Tyzzerella	0.9273	positive
**Ruminococcaceae_UCG-009**	Ruminiclostridium_1	0.6485	positive
**Ruminococcaceae_UCG-009**	[Eubacterium]_brachy_group	0.6828	positive
**Ruminococcaceae_UCG-009**	Tyzzerella_4	0.7435	positive
**Ruminococcaceae_UCG-009**	Blautia	0.6828	positive
**Ruminococcaceae_UCG-009**	Ruminiclostridium_5	0.8467	positive
**Enterococcus**	Oscillibacter	0.7091	positive
**Enterococcus**	Tyzzerella	0.7697	positive
**Enterococcus**	Ruminiclostridium_1	0.6364	positive
**Enterococcus**	Ruminiclostridium_5	0.7647	positive
**Lachnospiraceae_XPB1014_group**	Desulfovibrio	0.9586	positive
**Lachnospiraceae_XPB1014_group**	Pseudobutyrivibrio	0.8193	positive
**Lachnospiraceae_XPB1014_group**	Aerococcus	0.8621	negative
**Lachnospiraceae_XPB1014_group**	Glutamicibacter	0.8621	negative
**Lachnospiraceae_XPB1014_group**	Butyrivibrio_2	0.9586	positive
**Lachnospiraceae_XPB1014_group**	Alkalibacterium	0.8671	negative
**Lachnospiraceae_XPB1014_group**	Jeotgalicoccus	0.7788	negative
**Lachnospiraceae_XPB1014_group**	[Eubacterium]_brachy_group	0.7284	negative
**Lachnospiraceae_XPB1014_group**	Veillonellaceae_UCG-001	0.9469	positive
**Lachnospiraceae_XPB1014_group**	[Anaerorhabdus]_furcosa_group	0.9862	positive

**Lachnospiraceae_XPB1014_group**	Staphylococcus	0.8671	negative
**Lachnospiraceae_XPB1014_group**	Stenotrophomonas	0.7284	negative
**Lachnospiraceae_XPB1014_group**	Lachnospiraceae_ND3007_group	0.8959	positive
**Lachnospiraceae_XPB1014_group**	Solobacterium	0.7429	positive
**Lachnospiraceae_XPB1014_group**	Ruminococcus_2	0.9172	positive
**Lachnospiraceae_XPB1014_group**	Lachnospiraceae_NK3A20_group	0.9586	positive
**Lachnospiraceae_XPB1014_group**	Butyricimonas	0.9862	positive
**Lachnospiraceae_XPB1014_group**	Anaerovorax	0.9323	positive
**Lachnospiraceae_XPB1014_group**	Blautia	0.7284	negative
**Lachnospiraceae_XPB1014_group**	Corynebacterium_1	0.7134	negative
**Family_XIII_AD3011_group**	Lachnospiraceae_AC2044_group	0.8431	positive
**Family_XIII_AD3011_group**	Tyzzerella	0.8511	positive
**Family_XIII_AD3011_group**	Ruminiclostridium_1	0.8875	positive
**Family_XIII_AD3011_group**	Solobacterium	0.6643	positive
**Family_XIII_AD3011_group**	Ruminiclostridium_5	0.6438	positive
**Desulfovibrio**	Aerococcus	0.8621	negative
**Desulfovibrio**	Glutamicibacter	0.8621	negative
**Desulfovibrio**	Butyrivibrio_2	0.9586	positive
**Desulfovibrio**	Alkalibacterium	0.8338	negative
**Desulfovibrio**	Jeotgalicoccus	0.8574	negative
**Desulfovibrio**	[Eubacterium]_brachy_group	0.7284	negative
**Desulfovibrio**	Veillonellaceae_UCG-001	0.8667	positive

**Desulfovibrio**	[Anaerorhabdus]_furcosa_group	0.9448	positive
**Desulfovibrio**	Staphylococcus	0.8338	negative
**Desulfovibrio**	Stenotrophomonas	0.7284	negative
**Desulfovibrio**	Lachnospiraceae_ND3007_group	0.8158	positive
**Desulfovibrio**	Ruminococcus_2	0.931	positive
**Desulfovibrio**	Lachnospiraceae_NK3A20_group	0.9586	positive
**Desulfovibrio**	Butyricimonas	0.9448	positive
**Desulfovibrio**	Anaerovorax	0.8959	positive
**Desulfovibrio**	Blautia	0.7284	negative
**Desulfovibrio**	Ruminococcaceae_UCG-013	0.7079	positive
**Desulfovibrio**	Corynebacterium_1	0.8181	negative
**Odoribacter**	Parvibacter	0.6636	positive
**Odoribacter**	Corynebacterium_1	0.6442	positive
**Pseudobutyrivibrio**	Butyrivibrio_2	0.7238	positive
**Pseudobutyrivibrio**	Alkalibacterium	0.6924	negative
**Pseudobutyrivibrio**	Veillonellaceae_UCG-001	0.8653	positive
**Pseudobutyrivibrio**	[Anaerorhabdus]_furcosa_group	0.8193	positive
**Pseudobutyrivibrio**	Staphylococcus	0.6924	negative
**Pseudobutyrivibrio**	Lachnospiraceae_NK4A136_group	0.6386	positive
**Pseudobutyrivibrio**	Lachnospiraceae_ND3007_group	0.8653	positive
**Pseudobutyrivibrio**	Solobacterium	0.8653	positive
**Pseudobutyrivibrio**	Ruminococcus_2	0.6602	positive

**Pseudobutyrivibrio**	Lachnospiraceae_NK3A20_group	0.7238	positive
**Pseudobutyrivibrio**	Butyricimonas	0.8193	positive
**Pseudobutyrivibrio**	Anaerovorax	0.7645	positive
**Lachnospiraceae_AC2044_group**	Tyzzerella	0.6688	positive
**Lachnospiraceae_AC2044_group**	Ruminiclostridium_1	0.8406	positive
**Lachnospiraceae_AC2044_group**	Lachnospiraceae_NK4A136_group	0.7153	positive
**Lachnospiraceae_AC2044_group**	Solobacterium	0.6981	positive
**Aerococcus**	Glutamicibacter	0.9724	positive
**Aerococcus**	Butyrivibrio_2	0.8621	negative
**Aerococcus**	Alkalibacterium	0.8338	positive
**Aerococcus**	Jeotgalicoccus	0.8967	positive
**Aerococcus**	[Eubacterium]_brachy_group	0.7575	positive
**Aerococcus**	Veillonellaceae_UCG-001	0.7284	negative
**Aerococcus**	[Anaerorhabdus]_furcosa_group	0.8621	negative
**Aerococcus**	Gemmobacter	0.6443	positive
**Aerococcus**	Staphylococcus	0.9138	positive
**Aerococcus**	Stenotrophomonas	0.7356	positive
**Aerococcus**	Lachnospiraceae_ND3007_group	0.7284	negative
**Aerococcus**	Oxalobacter	0.867	positive
**Aerococcus**	Tyzzerella_4	0.7103	positive
**Aerococcus**	Ruminococcus_2	0.8621	negative
**Aerococcus**	Lachnospiraceae_NK3A20_group	0.8621	negative

**Aerococcus**	Butyricimonas	0.8621	negative
**Aerococcus**	Anaerovorax	0.7284	negative
**Aerococcus**	Blautia	0.7575	positive
**Glutamicibacter**	Butyrivibrio_2	0.8621	negative
**Glutamicibacter**	Alkalibacterium	0.8738	positive
**Glutamicibacter**	Jeotgalicoccus	0.8836	positive
**Glutamicibacter**	[Eubacterium]_brachy_group	0.6482	positive
**Glutamicibacter**	Veillonellaceae_UCG-001	0.7284	negative
**Glutamicibacter**	[Anaerorhabdus]_furcosa_group	0.8621	negative
**Glutamicibacter**	Gemmobacter	0.6602	positive
**Glutamicibacter**	Atopostipes	0.6628	positive
**Glutamicibacter**	Staphylococcus	0.9005	positive
**Glutamicibacter**	Stenotrophomonas	0.8303	positive
**Glutamicibacter**	Lachnospiraceae_ND3007_group	0.7284	negative
**Glutamicibacter**	Oxalobacter	0.8511	positive
**Glutamicibacter**	Ruminococcus_2	0.8621	negative
**Glutamicibacter**	Lachnospiraceae_NK3A20_group	0.8621	negative
**Glutamicibacter**	Butyricimonas	0.8621	negative
**Glutamicibacter**	Anaerovorax	0.7284	negative
**Glutamicibacter**	Blautia	0.6482	positive
**Butyrivibrio_2**	Alkalibacterium	0.9005	negative
**Butyrivibrio_2**	Jeotgalicoccus	0.8574	negative

**Butyrivibrio_2**	[Eubacterium]_brachy_group	0.7284	negative
**Butyrivibrio_2**	Veillonellaceae_UCG-001	0.8303	positive
**Butyrivibrio_2**	[Anaerorhabdus]_furcosa_group	0.9862	positive
**Butyrivibrio_2**	Staphylococcus	0.9005	negative
**Butyrivibrio_2**	Stenotrophomonas	0.7284	negative
**Butyrivibrio_2**	Methanobrevibacter	0.7133	positive
**Butyrivibrio_2**	Lachnospiraceae_ND3007_group	0.9323	positive
**Butyrivibrio_2**	Solobacterium	0.6774	positive
**Butyrivibrio_2**	Ruminococcus_2	0.9862	positive
**Butyrivibrio_2**	Lachnospiraceae_NK3A20_group	1.0	positive
**Butyrivibrio_2**	Butyricimonas	0.9862	positive
**Butyrivibrio_2**	Anaerovorax	0.8158	positive
**Butyrivibrio_2**	Blautia	0.7284	negative
**Butyrivibrio_2**	Ruminococcaceae_UCG-013	0.6602	positive
**Butyrivibrio_2**	Corynebacterium_1	0.805	negative
**Oscillibacter**	Tyzzerella	0.7697	positive
**Oscillibacter**	Parvibacter	0.6786	positive
**Tyzzerella**	Ruminiclostridium_1	0.7455	positive
**Tyzzerella**	Tyzzerella_4	0.7306	positive
**Tyzzerella**	Ruminiclostridium_5	0.7716	positive
**Alkalibacterium**	Jeotgalicoccus	0.7153	positive
**Alkalibacterium**	[Eubacterium]_brachy_group	0.6974	positive

**Alkalibacterium**	Veillonellaceae_UCG-001	0.7221	negative
**Alkalibacterium**	[Anaerorhabdus]_furcosa_group	0.9005	negative
**Alkalibacterium**	Staphylococcus	0.9484	positive
**Alkalibacterium**	Stenotrophomonas	0.7467	positive
**Alkalibacterium**	Lachnospiraceae_ND3007_group	0.8454	negative
**Alkalibacterium**	Parvibacter	0.6386	positive
**Alkalibacterium**	Ruminococcus_2	0.9005	negative
**Alkalibacterium**	Lachnospiraceae_NK3A20_group	0.9005	negative
**Alkalibacterium**	Butyricimonas	0.9005	negative
**Alkalibacterium**	Anaerovorax	0.6869	negative
**Alkalibacterium**	Blautia	0.6974	positive
**Alkalibacterium**	Corynebacterium_1	0.747	positive
**Ruminiclostridium_1**	Lachnospiraceae_NK4A136_group	0.6941	positive
**Ruminiclostridium_1**	Solobacterium	0.6623	positive
**Ruminiclostridium_1**	Ruminiclostridium_5	0.6418	positive
**Jeotgalicoccus**	[Anaerorhabdus]_furcosa_group	0.805	negative
**Jeotgalicoccus**	Gemmobacter	0.702	positive
**Jeotgalicoccus**	Staphylococcus	0.7913	positive
**Jeotgalicoccus**	Stenotrophomonas	0.712	positive
**Jeotgalicoccus**	Lachnospiraceae_ND3007_group	0.7189	negative
**Jeotgalicoccus**	Oxalobacter	0.8077	positive
**Jeotgalicoccus**	Ruminococcus_2	0.8574	negative

**Jeotgalicoccus**	Lachnospiraceae_NK3A20_group	0.8574	negative
**Jeotgalicoccus**	Butyricimonas	0.805	negative
**Jeotgalicoccus**	Anaerovorax	0.6497	negative
**Jeotgalicoccus**	Ruminococcaceae_UCG-013	0.6718	negative
**[Eubacterium]_brachy_group**	[Anaerorhabdus]_furcosa_group	0.7284	negative
**[Eubacterium]_brachy_group**	Staphylococcus	0.7256	positive
**[Eubacterium]_brachy_group**	Tyzzerella_4	0.7284	positive
**[Eubacterium]_brachy_group**	Parvibacter	0.8317	positive
**[Eubacterium]_brachy_group**	Ruminococcus_2	0.7284	negative
**[Eubacterium]_brachy_group**	Lachnospiraceae_NK3A20_group	0.7284	negative
**[Eubacterium]_brachy_group**	Butyricimonas	0.7284	negative
**[Eubacterium]_brachy_group**	Blautia	1.0	positive
**[Eubacterium]_brachy_group**	Corynebacterium_1	0.6981	positive
**[Eubacterium]_brachy_group**	Ruminiclostridium_5	0.7962	positive
**Veillonellaceae_UCG-001**	[Anaerorhabdus]_furcosa_group	0.8959	positive
**Veillonellaceae_UCG-001**	Staphylococcus	0.7221	negative
**Veillonellaceae_UCG-001**	Lachnospiraceae_ND3007_group	0.8115	positive
**Veillonellaceae_UCG-001**	Solobacterium	0.8115	positive
**Veillonellaceae_UCG-001**	Ruminococcus_2	0.7502	positive
**Veillonellaceae_UCG-001**	Lachnospiraceae_NK3A20_group	0.8303	positive
**Veillonellaceae_UCG-001**	Butyricimonas	0.8959	positive
**Veillonellaceae_UCG-001**	Anaerovorax	0.9846	positive

**[Anaerorhabdus]_furcosa_group**	Staphylococcus	0.9005	negative
**[Anaerorhabdus]_furcosa_group**	Stenotrophomonas	0.7284	negative
**[Anaerorhabdus]_furcosa_group**	Methanobrevibacter	0.6874	positive
**[Anaerorhabdus]_furcosa_group**	Lachnospiraceae_ND3007_group	0.9469	positive
**[Anaerorhabdus]_furcosa_group**	Solobacterium	0.7429	positive
**[Anaerorhabdus]_furcosa_group**	Ruminococcus_2	0.9586	positive
**[Anaerorhabdus]_furcosa_group**	Lachnospiraceae_NK3A20_group	0.9862	positive
**[Anaerorhabdus]_furcosa_group**	Butyricimonas	1.0	positive
**[Anaerorhabdus]_furcosa_group**	Anaerovorax	0.8667	positive
**[Anaerorhabdus]_furcosa_group**	Blautia	0.7284	negative
**[Anaerorhabdus]_furcosa_group**	Corynebacterium_1	0.7396	negative
**Gemmobacter**	Stenotrophomonas	0.7813	positive
**Atopostipes**	Stenotrophomonas	0.7962	positive
**Staphylococcus**	Lachnospiraceae_NK4A136_group	0.6903	negative
**Staphylococcus**	Lachnospiraceae_ND3007_group	0.8454	negative
**Staphylococcus**	Oxalobacter	0.7617	positive
**Staphylococcus**	Ruminococcus_2	0.9005	negative
**Staphylococcus**	Lachnospiraceae_NK3A20_group	0.9005	negative
**Staphylococcus**	Butyricimonas	0.9005	negative
**Staphylococcus**	Anaerovorax	0.6869	negative
**Staphylococcus**	Blautia	0.7256	positive
**Staphylococcus**	Corynebacterium_1	0.7216	positive

**Lachnospiraceae_NK4A136_group**	Lachnospiraceae_ND3007_group	0.7256	positive
**Lachnospiraceae_NK4A136_group**	Oxalobacter	0.6924	negative
**Stenotrophomonas**	Parvibacter	0.7477	positive
**Stenotrophomonas**	Ruminococcus_2	0.7284	negative
**Stenotrophomonas**	Lachnospiraceae_NK3A20_group	0.7284	negative
**Stenotrophomonas**	Butyricimonas	0.7284	negative
**Methanobrevibacter**	Lachnospiraceae_ND3007_group	0.815	positive
**Methanobrevibacter**	Solobacterium	0.6712	positive
**Methanobrevibacter**	Ruminococcus_2	0.7004	positive
**Methanobrevibacter**	Lachnospiraceae_NK3A20_group	0.7133	positive
**Methanobrevibacter**	Butyricimonas	0.6874	positive
**Lachnospiraceae_ND3007_group**	Solobacterium	0.8115	positive
**Lachnospiraceae_ND3007_group**	Ruminococcus_2	0.9032	positive
**Lachnospiraceae_ND3007_group**	Lachnospiraceae_NK3A20_group	0.9323	positive
**Lachnospiraceae_ND3007_group**	Butyricimonas	0.9469	positive
**Lachnospiraceae_ND3007_group**	Anaerovorax	0.7423	positive
**Lachnospiraceae_ND3007_group**	Corynebacterium_1	0.6567	negative
**Tyzzerella_4**	Blautia	0.7284	positive
**Tyzzerella_4**	Ruminiclostridium_5	0.7429	positive
**Solobacterium**	Lachnospiraceae_NK3A20_group	0.6774	positive
**Solobacterium**	Butyricimonas	0.7429	positive
**Solobacterium**	Anaerovorax	0.7423	positive

**Parvibacter**	Blautia	0.8317	positive
**Ruminococcus_2**	Lachnospiraceae_NK3A20_group	0.9862	positive
**Ruminococcus_2**	Butyricimonas	0.9586	positive
**Ruminococcus_2**	Anaerovorax	0.7356	positive
**Ruminococcus_2**	Blautia	0.7284	negative
**Ruminococcus_2**	Ruminococcaceae_UCG-013	0.7398	positive
**Ruminococcus_2**	Corynebacterium_1	0.805	negative
**Lachnospiraceae_NK3A20_group**	Butyricimonas	0.9862	positive
**Lachnospiraceae_NK3A20_group**	Anaerovorax	0.8158	positive
**Lachnospiraceae_NK3A20_group**	Blautia	0.7284	negative
**Lachnospiraceae_NK3A20_group**	Ruminococcaceae_UCG-013	0.6602	positive
**Lachnospiraceae_NK3A20_group**	Corynebacterium_1	0.805	negative
**Butyricimonas**	Anaerovorax	0.8667	positive
**Butyricimonas**	Blautia	0.7284	negative
**Butyricimonas**	Corynebacterium_1	0.7396	negative
**Blautia**	Corynebacterium_1	0.6981	positive
**Blautia**	Ruminiclostridium_5	0.7962	positive
**Ruminococcaceae_UCG-013**	Corynebacterium_1	0.6718	negative
